# Greener Synthesis of Antiproliferative Furoxans via Multicomponent Reactions

**DOI:** 10.3390/molecules27061756

**Published:** 2022-03-08

**Authors:** Mariana Ingold, Victoria de la Sovera, Rosina Dapueto, Paola Hernández, Williams Porcal, Gloria V. López

**Affiliations:** 1 Laboratorio de Desarrollo de Fármacos y Biología Vascular, Institut Pasteur Montevideo, Mataojo 2020, Montevideo 11400, Uruguay; mingold@pasteur.edu.uy (M.I.); vdelasovera@fq.edu.uy (V.d.l.S.); 2 Departamento de Química Orgánica, Facultad de Química, Universidad de la República, Av. General Flores 2124, Montevideo 11800, Uruguay; 3 I + D Biomédico, Centro Uruguayo de Imagenología Molecular, Montevideo 11600, Uruguay; dapuetor@gmail.com; 4 Departamento de Genética, Instituto de Investigaciones Biológicas Clemente Estable, Montevideo 11600, Uruguay; paohernz@gmail.com

**Keywords:** multicomponent reactions, furoxans, green synthesis, nitric oxide donor, antiproliferative activity

## Abstract

Prostate and bladder cancers are commonly diagnosed malignancies in men. Several nitric oxide donor compounds with strong antitumor activity have been reported. Thus, continuing with our efforts to explore the chemical space around bioactive furoxan moiety, multicomponent reactions were employed for the rapid generation of molecular diversity and complexity. We herein report the use of Ugi and Groebke–Blackburn–Bienaymé multicomponent reactions under efficient, safe, and environmentally friendly conditions to synthesize a small collection of nitric-oxide-releasing molecules. The in vitro antiproliferative activity of the synthesized compounds was measured against two different human cancer cell lines, LNCaP (prostate) and T24 (bladder). Almost all compounds displayed antiproliferative activity against both cancer cell lines, providing lead compounds with nanomolar GI_50_ values against the cancer bladder cell line with selectivity indices higher than 10.

## 1. Introduction

Cancer is one of the main causes of death worldwide, accounting for nearly 10 million deaths in 2020 [1]. Bladder cancer is the fourth most common cancer in men. It is a complex disease associated with high rates of morbidity and mortality if not treated optimally. The normal treatment is transurethral resection with chemoradiation, but for some patients first-line chemotherapy cannot control the disease [2]. Prostate cancer is the second most frequent malignancy in men worldwide. Despite advances in knowledge about this disease, an effective therapeutic agent for its treatment is still needed [3]. For these reasons, it is necessary to develop new, safe, and more efficient compounds for the treatment of these pathologies.

Currently one of the key objectives in medicinal chemistry is the search for new drugs following the Green Chemistry guidelines [4,5]. 

In this context, our group has dedicated the last years to the search for compounds with antitumoral activity via efficient and more environmentally friendly methodologies. A synthetic tool explored by our group is multicomponent reactions (MCR). MCR are those reactions in which the product is formed from three or more reagents in a single reaction step with high atomic efficiency [6]. These reactions are excellent tools in medicinal chemistry for the generation of structural diversity and for hybridizing/conjugating different pharmacophores in the same molecule, hence the importance of their development for our research group [7]. In this sense, we have previously described a green process for the synthesis of tocopherol analogues via Passerini and Ugi MCR and their antiproliferative activity against a panel of six human solid tumor cell lines [8]. Then, a second-generation focused library of nitric-oxide-releasing compounds was prepared by microwave-assisted Passerini and Ugi reactions. Nearly all compounds displayed potent antiproliferative activities against a panel of human solid tumor cell lines, with compound Ugi-Fx (**8a**) (Table 1) exhibiting the strongest activity on the SW1573 lung cell line (GI_50_ = 21 nM) with a selectivity index of 470. Preliminary mechanistic studies suggest a relationship between nitric oxide (·NO) release and antiproliferative activity [9]. 

**Figure 1:**
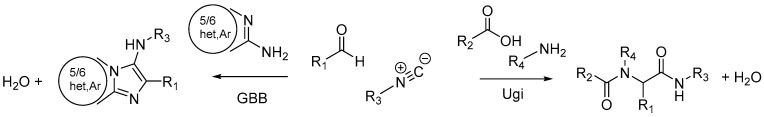
Ugi and GBB reactions.

Among the MCRs, the Ugi and the Groebke–Blackburn–Bienaymé (GBB) reactions have been widely used in medicinal chemistry. The Ugi four-component reaction was first reported in 1959 by Ivar Ugi and is one of the most important MCRs that allows access to peptide-mimetic structures [10]. This reaction combines an amine, an aldehyde, a carboxylic acid, and an isocyanide to form a bis-amide derivative (Figure 1). In 1998, a new variant of the Ugi MCR was independently reported by three researchers from different countries (Katrin Groebke (Switzerland), Christopher Blackburn (USA), and Hugues Bienaymé (France)), named the Groebke–Blackburn–Bienaymé reaction [11,12,13]. This three-component reaction involves an aldehyde, a 2-aminoazine, and an isocyanide, giving a place to a highly substituted and fused imidazole derivative (Figure 1). Both MCRs are great instruments for drug development [14,15], and they have been explored under green chemistry conditions [8,9,16,17,18]. Previous studies showed that peptidomimetic structures and imidazoheterocycles obtained by Ugi and GBB multicomponent reactions, respectively, present promising antitumor activities in various models [15,19,20].

To continue in the line of our previous work, we designed a third series of compounds with the ability to release ·NO, particularly incorporating the furoxan group. Nitric oxide, ·NO, is a free radical that was described by Nobel Prize winners Furchgott, Ignarro, and Murad as a signaling molecule in the cardiovascular system. Since then, a wide variety of biological processes in which this compound participates have been described in addition to its application in the area of drug discovery [21]. The role of ·NO in cancer has been extensively studied. It has been determined that its action is variable, sometimes as an antineoplastic agent and others as a proneoplastic agent. This depends mainly on its intracellular concentration but also on the microenvironment, cell type, and the exposure time to ·NO. Some studies suggest that at high levels this radical can exert its antitumor activity through various mechanisms [22,23]. Among the structures capable of releasing ·NO, furoxans are a widely used example in medicinal chemistry [24], especially in the search for anticancer agents [25]. Antitumoral drug discovery efforts have registered an increased application of the molecular hybridization concept in the last years. Several hybrid compounds containing ·NO donor moieties have been reported with significant antiproliferative activity [2,26,27]. In this context, we designed a molecular hybridization strategy combining Ugi and GBB products with the furoxan system as a ⋅NO-releasing group.

In summary, in this work we report the use of Ugi and Groebke–Blackburn–Bienaymé multicomponent reactions under efficient, safe, and ecofriendly conditions to synthesize a small collection of nitric-oxide-releasing molecules, using the furoxan system as a pharmacophore and their evaluation as antiproliferative agents in bladder and prostate cancer cells.

## 2. Results and Discussion

### 2.1. Chemistry

#### 2.1.1. Ugi Reaction

**Scheme 1:**
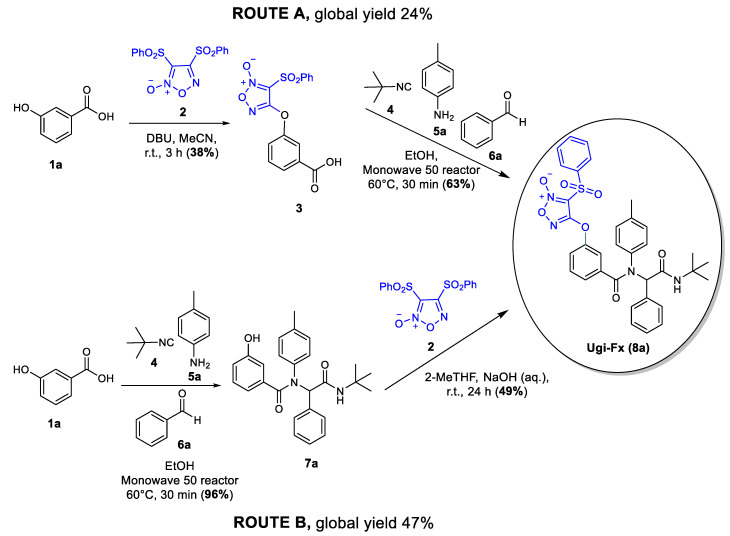
Explored routes for the synthesis of Ugi-Fx (**8a**).

In our previous work, we reported the synthesis of the compound Ugi-Fx (**8a**) according to route A (Scheme 1), where 3,4-bis(phenylsulfonyl)furoxan (**2**) was previously coupled to 3-hydroxybenzoic acid to yield compound **3**. Then, the intermediate **3** was used as a component in the Ugi reaction, obtaining the desired product **8a** in an overall yield of 24% [9]. At the beginning of the present work, we decided to study the synthetic strategy according to route B (Scheme 1). In the first step, the Ugi reaction was carried out in an excellent yield, and in the second step, 3,4-bis(phenylsulfonyl)furoxan (**2**) was linked to Ugi structure **7a**, which led to an improvement of almost double the overall yield, obtaining Ugi-Fx (**8a**) in a 47% yield. It is noteworthy that greener conditions were used in both reaction steps, using green solvents under mild and efficient heating conditions.

The Ugi reactions were carried out using a Monowave 50 reactor (Anton Paar). It employs conductive heating of a sealed glass vessel via a stainless-steel heating jacket and includes online temperature and pressure monitoring in addition to magnetic stirring. In this sense, the results obtained using a microwave reactor can be replicated in the simpler, cheaper, and smaller synthesis reactor [28]. For example, the reaction to obtain Ugi-Fx (**8a**) from compound **3**, was previously carried out in a microwave reactor [9], and the yield was the same using the Monowave 50 reactor (63%).

For the coupling between the phenol intermediate **7a** and furoxan **2**, we decided to look for greener conditions than those previously used. Therefore, the reaction was carried out using 2-MeTHF as a greener solvent (biorenewable) [29] and NaOH as an environmentally friendly base [30].

**Figure 2:**
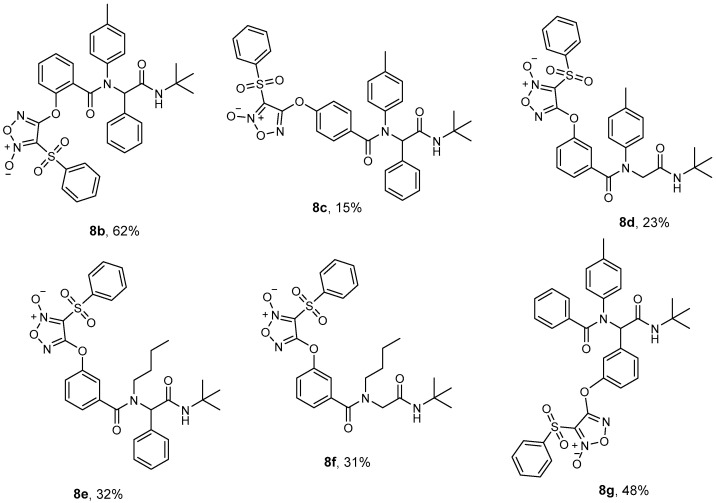
Scope of ·NO donors via Ugi MCR under optimized greener conditions (overall yield).

Once we optimized the synthesis of the lead compound Ugi-Fx (**8a**), we varied the different components of the reaction to obtain new derivatives and expand the chemical space studied. We changed the relative position of the furoxan group on the aromatic ring (Figure 2, **8b** and **8c**), the amine and aldehyde from aromatic to aliphatic (Figure 2, **8d**–**f**), and we also changed the furoxan group from the carboxylic acid component to the aldehyde component (Figure 2, **8g**) using the reaction conditions of route B. For both reaction steps, we obtained moderate to very good yields.

#### 2.1.2. GBB Reaction

**Scheme 2:**
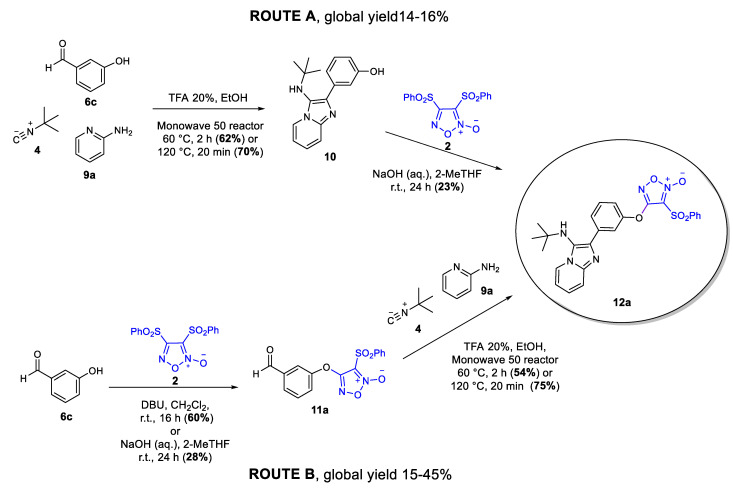
Synthetic routes explored for the synthesis of GBB derivative **12a**.

To broaden the chemical diversity even further, we decided to synthesize hybrid compounds incorporating the furoxan group into structures derived from the GBB MCR. First, we studied the optimal synthesis pathway for the desired compounds, considering the synthetic methodologies used to obtain Ugi-Fx (**8a**, Scheme 1). The synthetic procedure for the GBB target compounds was initially studied for the product **12a**. We explored a route analogous to the one optimized for Ugi-Fx (**8a**), (route A, Scheme 2), with the multicomponent reaction as a first step and subsequent coupling with the furoxan system. The GBB reaction was performed using 3-hydroxybenzaldehyde (**6c**) as the aldehyde component, *tert*-butyl isocyanide, and 2-aminopyridine under greener conditions. Catalytic amounts of TFA (20 mol%) in EtOH and mild temperature conditions were used in the Monowave 50 reactor to obtain GBB product **10** with a good yield. When this reaction was carried out by heating at 120°C for 20 min, a slightly improved yield was observed. Phenol **10** was then coupled to 3,4-bis(phenylsulfonyl)furoxan (**2**) in a subsequent step using NaOH and 2-MeTHF to give the desired compound **12a** in low yield. Based on this result, we decided to explore derivative **12a** synthesis using another route (route B, Scheme 2) by reversing the steps of route A (Scheme 2). Aldehyde intermediate **11a** was obtained in 28% yield under greener conditions (NaOH and 2-MeTHF, 24 h). Due to this low yield, the reaction was also assayed according to the procedure described in the literature [8], obtaining **11a** in 60% yield. In the second step, the GBB reaction was carried out under the same conditions described in route A. When the reaction was heated at 60 °C for 2 h, product **12a** was obtained in a 54% yield after precipitating in the crude reaction mixture. On the other hand, when the reaction was performed at higher temperatures (120 °C) and shorter times (20 min) a higher yield was obtained for the desired GBB derivative **12a** (75%). In this case, **12a** did not precipitate in the crude reaction mixture, and column chromatography purification was needed.

It is well-known that the GBB reaction could follow two possible pathways leading to two regioisomers, but the amidine building blocks used in the present work usually tend to form only one regioisomer [15]. The presence of the most common regioisomer in GBB derivatives **10** and **12a** was evidenced by the 2D-NOESY-NMR (see Supporting Information).

**Scheme 3:**
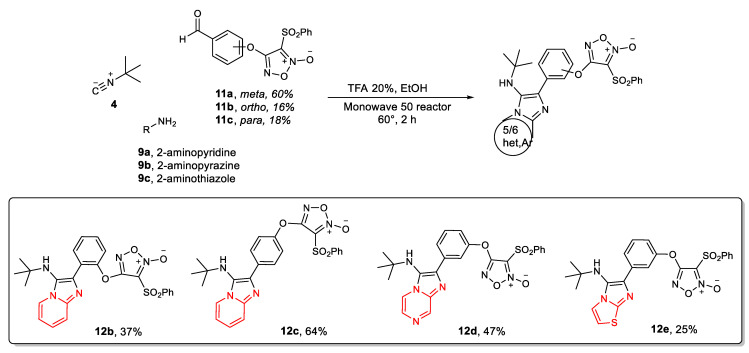
Synthesis of the GBB-furoxan compounds **12b**–**12d** under green conditions.

Analyzing the results in both synthetic pathways, route B was chosen for the synthesis of the GBB-furoxan derivatives **12b**–**12e** (Scheme 3). Classical conditions were selected for the intermediate aldehyde synthesis. Mild temperatures were chosen for the GBB reaction, considering the greener purification procedure. Under these reaction conditions, route B gave a higher global yield, 32% versus 14–16% for route A. 

Scheme 3 shows the results obtained in the GBB reaction for each aldehyde-furoxan and 2-aminoazine employed. To synthesize aldehydes **11b** and **11c**, as for **11a**, classical conditions and greener conditions were tested, obtaining higher yield using classical ones (16 % vs. 5%, 18% vs. 2%) [31]. As can be observed for the aldehydes substituted in the ortho and para positions, coupling with furoxan derivative **2** gave lower yields (16 and 18%, respectively, Scheme 3). The meta-substituted compound (**12a**) gave the best global yield, followed by the para-substituted (**12c**), and, finally, the ortho-substituted (**12b**) (32, 12 and 6% correspondingly).

To widen the scope of the GBB reaction, we explored other amidines, maintaining the isocyanide and aldehyde **11a**, which showed the best performance. For this purpose, 2-aminopirazine and 2-aminothiazole were used to obtain the corresponding products **12d** and **12e** in 47 and 25% yields, respectively (Scheme 3). It is noteworthy that this is the first time that a GBB reaction is described using furoxan derivatives.

### 2.2. Biology

#### 2.2.1. Antiproliferative Activity

In vitro antiproliferative activity of the compounds was determined in cells derived from bladder cancer (T24) and prostate cancer (LNCaP). The cells were incubated in the presence of the compounds for 48 h. The antiproliferative activity was determined using the sulforhodamine B assay [32]. The results were expressed as GI_50_, which is the drug concentration resulting in a 50% reduction of cellular net growth when compared with the values of untreated control cells. The standard anticancer drug cisplatin was used as a positive control, and furoxan **3** and **11a** were also included as ⋅NO donor reference compounds. The results are summarized in Table 2 for the Ugi product **7a**, Ugi-Fx (**8a**) and **8b**–**g** Ugi-furoxan derivatives, and GBB product **10** and **12a**–**e** GBB-furoxan derivatives. 

The results show that every compound containing the coupled furoxan moiety exhibited better antiproliferative activity than the cisplatin and furoxan reference compounds (**3** and **11a**) in the T24 bladder cancer cell line (Table 2). Compounds Ugi-Fx (**8a**), **12b** and **12e** stand out, with GI_50_ values in the sub-micromolar range. On the other hand, most compounds in the prostate cancer cell line LNCaP were less active, except for compounds **12b** and **12d,** which displayed GI_50_ values two times lower than the drug reference cisplatin and three to four times lower than furoxan **3** and **11a**. When analyzing the antiproliferative activity of Ugi derivatives **8a**–**8g** on the bladder cancer cell line, we can conclude that the different structural changes introduced did not improve the activity of the leader Ugi-Fx (**8a**). On the same cancer cell line (T24), the GBB derivatives **12a**–**e** also did not overcome the leader’s activity Ugi-Fx (**8a**), although compounds **12b** and **12e** displayed a similar GI_50_. The structure–activity relationship (SAR) study revealed that modifications in Ugi products were not a critical factor for modulating the activity, although there are some points to be considered. The change in the relative position of the furoxan group on the acid component (**8b** and **8c** derivatives, Figure 2) produces a two- to three-fold decrease relative to the parent compound Ugi-Fx (**8a**) on the antiproliferative activity in the T24 cell line (Table 2). However, the same order activity was observed for all three compounds on the LNCaP cell line. The substitution of the aryl group of Ugi-Fx (**8a**) by hydrogen in the aldehyde component (derivatives **8d** and **8f**) and/or by an alkyl group in the amine component (derivatives **8e** and **8f**) produced a decrease in activity, mainly in the bladder cancer line (Table 2). A similar response was observed when the furoxan group was introduced into the Ugi structure via the aldehyde component (**8g**). In the GBB group of compounds, the presence of a furoxan group in the ortho position of the substituent (**12b**) improved antiproliferative activity in both tumor cell lines (derivatives **12a**–**c**, Table 2). In fact, **12b**, an imidazo[1,2-a]pyridine derivative, was approximately three to four times more active than the anticancer drug cisplatin. The GBB compounds **12d** and **12e**, which incorporate an imidazo[1,2-a]pyrazine and imidazo[2,1-b]thiazole system, showed similar GI_50_ values when compared to **12b** for the LNCaP and T24 tumor cell line, respectively. It is worth mentioning that compounds **7a** and **10**, derived from the reaction of Ugi and GBB, respectively, which do not contain the coupled furoxan moiety, were inactive (GI_50_ > 100 µM) on both cell lines studied.

#### 2.2.2. Selectivity Index

As a first approximation of the safety profile of the synthesized compounds and to determine the cytotoxic selectivity against cancer cells, the antiproliferative activity in non-cancer cells was studied. This evaluation was carried out in human keratinocyte HaCaT cells with 0.4–100 µM of the test compounds using the sulforhodamine B assay. The selectivity index (SI) was calculated for these compounds by dividing the GI_50_ for HaCaT by the GI_50_ for each cancer cell line. The results presented in Table 3 show that all compounds were selective toward T24 cancerous cell, showing better selectivity than the reference compound cisplatin. The derivatives Ugi-Fx (**8a**) and **12e** stand out, with selectivity indices of 14.5 and 10.8, respectively. These compounds exhibited much better selectivity than the furoxan derivative **2** and the furoxan reference compounds **3** and **11a**, confirming that the conjunction with a structure derived from MCRs improves the activity as well as the toxicity. Concerning the LNCaP prostate cancer cell line, as for the reference drug, no selectivity was observed. Despite these results being obtained in an unspecific mammalian cytotoxicity model, it is interesting to note that imidazo[1,2-a]pyrazine **12d** presented an SI value 10 times higher than the reference drug cisplatin (SI 1.2 vs. 0.1). Therefore, this new GBB-furoxan derivative can be a starting point in the design of new structures with improved selectivity.

#### 2.2.3. Assessment of ·NO Release

**Figure 3:**
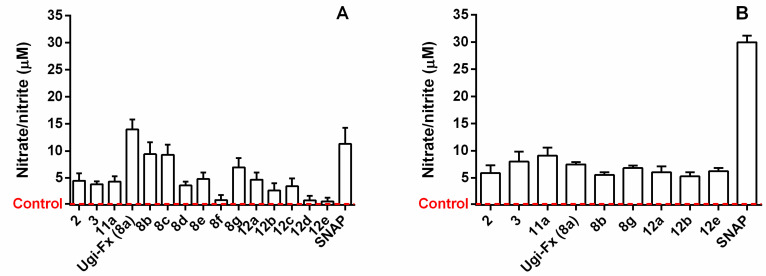
Levels of ·NO produced by Ugi-furoxan and GBB-furoxan derivatives (**A**) in T24 cells and (**B**) in LNCaP cells. The results are the means ± SD of three independent experiments. SNAP is the ·NO-releasing positive control compound. The red dotted line indicates the baseline, corresponding to the negative control that was treated only with culture medium.

The compounds developed in this work contain the furoxan moiety as an antitumor pharmacophore since it is well known that it is capable of releasing high amounts of ·NO through a thiol-dependent mechanism [34]. In this context, the ·NO production capacity of these derivatives was determined in physiological solution and in T24 and LNCaP supernatants using the Griess assay. The results are presented as nitrite production in Table S1 and Figure 3. The furoxan precursors **2**, **3**, and **11a** were included for comparison, and the Ugi derivative **7a** and GBB product **10** were included as negative controls.

The ability to release ·NO in physiological solution was determined by the Griess reaction after incubation for 1 h and 3 h at 37 °C in a pH 7.4 buffered water-methanol mixture in the presence of L-cysteine (5-fold molar excess). The results depicted in Table S1 show that all new furoxans were able to induce nitrite formation at levels ranging from 13.50% to 38.45%. Although the furoxan ring is present in all compounds substituted at position 3 by a phenylsulfonyl group, some differences were observed. In general, Ugi derivatives produced higher NO levels than GBB derivatives, except for **12b**, which exhibited comparable NO-releasing activity.

The ·NO production was also measured in T24 cell supernatants (Figure 3A) and LNCaP supernatants (Figure 3B) exposed to 100 µM of each compound for 3 h, which was defined as the optimal time point to measure this species by furoxan-derived compounds according to our previous studies [33,35]. Almost every compound was capable of releasing several levels more ·NO than the control cell medium, both in the bladder cancer cells and in the prostate cancer cells. An analysis of the data of both assessments also showed that the NO-releasing capacity of the products in cell milieu differs from that in physiological conditions, suggesting that they present different physicochemical properties and reactivity towards thiols in the cellular environment (Table S1 and Figure 3).

**Figure 4:**
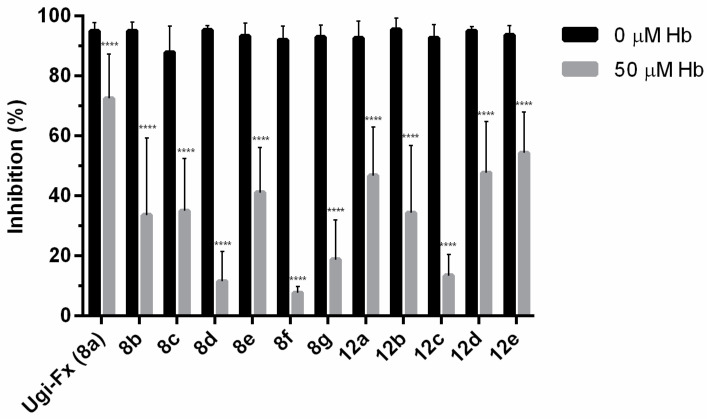
Effects of hemoglobin (Hb) on the antiproliferative effect of selected ·NO-donor compounds. The results are expressed as the percentage of cell growth inhibition relative to the control cells. The data are the means ± SD obtained from three determinations. Statistical analysis: a two-way ANOVA followed by a Bonferroni multiple comparison test: **** *p* < 0.0001.

In this context, we next decided to study the effect of ·NO on their antiproliferative activity against T24 cells after incubation with an ·NO scavenger (Hb, hemoglobin) [36]. For this purpose, T24 cells were pretreated for 1 h with 0 or 50 µM of Hb and then the ·NO-donor compounds were added at 10 µM for 48 h. As seen in Figure 4, the compounds markedly inhibited the growth of T24 cells in the absence of Hb, but this antiproliferative effect was decreased by pretreatment with Hb. These results indicate that the release of nitric oxide is involved in the antiproliferative activity against cancer cells. However, while for some compounds, such as **8d**, ·NO release seems to be crucial for the antiproliferative activity, for others, such as Ugi-Fx (**8a**), other mechanisms would account for its anticancer effect. It is well-known that the action of 3-phenylsulfonylfuroxan derivatives is connected to ⋅NO release due to their interaction with free intracellular thiols or thiol groups of proteins. However, this action may also involve a covalent bond to thiol biomolecules with the concomitant displacement of the phenylsulfonyl group, leading to a cytotoxic adduct accumulation in the cell milieu [37,38,39,40]. This fact should explain that some compounds described herein present a significant antiproliferative activity but a low NO release (i.e., **12b** and **12e**) or high antiproliferative activity even in the presence of a ⋅NO scavenger (Ugi-Fx **8a**). Further studies to elucidate the possible mechanisms of action involved will be performed. 

## 3. Materials and Methods

### 3.1. Chemistry

#### 3.1.1. General Experimental Information

The mass spectra (MS) were recorded on a Shimadzu GC–MS QP 1100 EX instrument using the electron impact mode (70 eV). A Thermo Scientific Q Exactive Hybrid Quadrupole-Orbitrap Mass Spectrometer was employed for high-resolution mass analysis using MeOH as a solvent. The NMR spectra were obtained in different deuterated solvents on a Bruker Avance DPX-400 instrument. The proton chemical shifts (δ) are reported in ppm downfield from TMS as an internal reference, and the carbon chemical shifts are reported in ppm relative to the center line of the corresponding solvent. Analytical TLC was performed on silica gel 60F-254 plates and was visualized with UV light (254 nm) and/or vanillin in an acidic ethanolic solution. Flash column chromatography was performed using silica gel (Kieselgel 60, EM reagent, 230–400 mesh). The chemicals and reagents were purchased from Sigma-Aldrich and were used as received. All GBB and Ugi reactions were carried out in a commercial Anton Paar Monowave 50 reactor using 10 mL borosilicate glass vials with silicone caps. The temperature was monitored by an external contact sensor placed at the cavity bottom and measuring the surface temperature of the reaction vessel.

#### 3.1.2. Experimental Procedures and Characterization Data for the Compounds

General procedure for Ugi products (**7a**–**7g**). Method A (aromatic amine). A glass tube was charged sequentially with aldehyde (1.0 mmol), amine (1.0 mmol), acid (1.0 mmol), isocyanide (1.0 mmol), and 1.5 mL EtOH. The sealed test tube was heated for 30 min at 60 °C using Monowave 50 reactor. The vial was allowed to cool, and the EtOH was evaporated under reduced pressure. The residue was purified by flash column chromatography (SiO_2_ and Hexane/EtOAc). Method B (aliphatic amine). A glass tube was charged with aldehyde (1.0 mmol), amine (1.0 mmol), and 1.0 mL EtOH and the reaction mixture was magnetically stirred for 3 h at room temperature. Subsequently, the acid (1.0 mmol), the isocyanide (1.0 mmol), and 0.5 mL EtOH were added. The sealed test tube was heated for 30 min at 60 °C using Monowave 50 reactor. The vial was allowed to cool, and the EtOH was evaporated under reduced pressure. The residue was purified by flash column chromatography (SiO_2_ and Hexane/EtOAc).

*N-(1-(tert-butylaminocarbonyl)-1-phenylmethyl)-N-(4-methylphenyl)-3-hydroxyphenyl carboxamide* (**7a**). This compound was obtained by method A. The crude product was purified by flash column chromatography (hexane/ EtOAc, 8/2) to render the desired product as a white solid, m.p. (98–100) °C, yield 96%. ^1^H-NMR (400 MHz, CDCl_3_) *δ* 7.31–7.25 (m, 5H); 7.18 (s, 1H); 7.07–7.06 (m, 1H); 6.96–6.88 (m, 3H); 6.81–6.79 (m, 2H); 6.74–6.69 (m, 2H); 5.97 (s, 1H); 5.87 (s, 1H); 2.16 (s, 3H); and 1.35 (s, 9H). ^13^C-NMR (101 MHz, CDCl_3_) *δ* 171.4; 168.8; 156.0; 138.6; 137.2; 136.9; 134.9; 130.0; 129.6; 129.0; 128.8; 128.5; 128.4; 120.2; 117.0; 115.8; 67.8; 51.7; 28.6; and 21.0. MS (IE, 70 eV) *m*/*z* (%): 344 (M^+^-NH *t*-butyl, 0.5); 316 (18); 195 (6); 121 (100); 106 (3); 91 (10); 77 (1); and 57 (4). HRMS (ESI+): *m*/*z* calculated for C_26_H_28_N_2_O_3_Na 439.1998 [M+Na]^+^, found 439.1999.

*N-(1-(tert-butylaminocarbonyl)-1-phenylmethyl)-N-(4-methylphenyl)-2-hydroxyphenyl carboxamide* (**7b**). This compound was obtained by method A. The crude product was purified by flash column chromatography (hexane/EtOAc, 8/2) to render the desired product as a white solid, m.p. (179–181) °C, yield 89%. ^1^H-NMR (400 MHz, CDCl_3_) *δ* 7.28 (s, 5H), 7.15–7.11 (m, 1H), 6.96–6.84 (m, 6H), 6.49–6.45 (m, 1H), 6.06 (s, 1H), 5.72 (s, 1H), 2.22 (s, 3H), and 1.39 (s, 9H). ^13^C-NMR (101 MHz, CDCl_3_) *δ* 171.4, 168.8, 158.5, 138.6, 137.4, 134.5, 132.2, 130.1, 129.6, 129.4, 129.0, 128.6, 128.5, 118.1, 117.4, 68.2, 52.0, 28.6, and 21.0. MS (IE, 70 eV) *m*/*z*(%): 416 (0.8); 317 (15); 296 (13); 196 (100); 121 (23); 93 (6); and 91(16). HRMS (FAB): *m*/*z* [M+Na]^+^ calculated for C_26_H_28_N_2_O_3_Na: 439.1998, found: 439.1996.

*N-(1-(tert-butylaminocarbonyl)-1-phenylmethyl)-N-(4-methylphenyl)-4-hydroxyphenyl carboxamide* (**7c**). This compound was obtained by method A. The crude product was purified by flash column chromatography (hexane/ EtOAc, 7/3) to render the desired product as a white solid, m.p. (99–101) °C, yield 47%. ^1^H-NMR (400 MHz, CDCl_3_) *δ* 7.53 (s, 1H), 7.32–7.25 (m, 5H), 7.15 (d, *J* = 8.7 Hz, 2H), 6.87–6.82 (m, 4H), 6.57 (d, *J* = 8.7 Hz, 2H), 5.90 (bs, 2H), 2.19 (s, 3H), and 1.35 (s, 9H). ^13^C-NMR (101 MHz, CDCl_3_) *δ* 171.1, 169.1, 158.0, 139.6, 136.7, 135.1, 131.2, 129.8, 129.3, 129.2, 128.5, 128.3, 127.0, 114.7, 68.7, 51.7, 28.6, and 21.0. MS (IE, 70 eV) *m*/*z*(%): 417(0.2); 317(29); 295(0.5); 196 (100); 121 (48); and 91 (13). HRMS (ESI+): *m*/*z* calculated for C_26_H_28_N_2_O_3_: 416.2100 [M]^+^, found: 417.2185.

*N-(1-(tert-butylaminocarbonyl)methyl)-N-(4-methylphenyl)-3(hydroxy)phenylcarbox amide* (**7d**). This compound was obtained by method A. The crude product was purified by flash column chromatography (hexane/EtOAc, 1/1) to render the desired product as a white solid, m.p. (196–198) °C, yield 38%. ^1^H-NMR (400 MHz, acetone-d_6_) *δ* 8.42 (s, 1H), 7.12–7.10 (m, 2H), 7.06–7.00 (m, 3H), 6.90–6.89 (m, 1H), 6.80–6.73 (m, 2H), 4.38 (s, 2H), 2.25 (s, 3H), and 1.34 (s, 9H). ^13^C-NMR (101 MHz, acetone-d_6_) *δ* 170.0, 167.5, 156.7, 142.0, 137.9, 136.0, 129.3, 128.6, 127.2, 119.7, 116.3, 115.6, 54.0, 50.5, 28.1, and 20.0. MS (IE, 70 eV) *m*/*z*(%): 340 (4); 268 (1); 240 (18); 202 (11); 146 (53): 121 (100); 93 (18); and 91 (6). HRMS (ESI+): *m*/*z* calculated for C_20_H_24_N_2_O_3_: 340.1787 [M]^+^, found: 341.1864.

*N-(1-(tert-butylaminocarbonyl)-1-phenylmethyl)-N-(butyl)-3-hydroxyphenylcarbox amide* (**7e**). This compound was obtained by method B. The crude product was purified by flash column chromatography (hexane/ EtOAc, 7/3) to render the desired product as a white solid, m.p. (179–181) °C, yield 77%. ^1^H-NMR (400 MHz, acetone-d_6_) *δ* 8.63 (s, 1H), 7.41–7.35 (m, 5H), 7.30–2.26 (m, 1H), 6.92–6.88 (m, 3H), 5.51 (bs, 1H), 3.51–3.27 (m, 2H), 1.46–1.31 (m, 1H), 1.37 (s, 9H), 1.03–0.82 (m, 3H), and 0.63–0.53 (m, 3H). ^13^C-NMR (101 MHz, acetone-d_6_) *δ* 171.4, 169.0, 157.4, 139.1, 137.1, 129.6, 129.2, 128.4, 127.9, 117.4, 116.0, 113.4, 71.0, 50.8, 45.0, 30.4, 27.9, 19.9, and 12.9. MS (IE, 70 eV) *m*/*z*(%): 382 (0.2); 310 (0.5); 282 (33); 261 (0.6); 121 (100); 93 (13); and 91 (5). HRMS (ESI+): *m*/*z* calculated for C_23_H_30_N_2_O_3_: 382.2256 [M]^+^, found: 383.2341.

*N-(1-(tert-butylaminocarbonyl)methyl)-N-(butyl)-3-(hydroxy)phenylcarboxamide* (**7f**). This compound was obtained by method B. The crude product was purified by flash column chromatography (hexane/EtOAc, 1/1) to render the desired product as a white solid, m.p. (131–133) °C, yield 84%. Due to the tautomeric equilibrium, the signals of the compound in the ^1^H-NMR are duplicated at room temperature (298 K). When heating to 348 K, the phenomenon of coalescence is observed, and there is a signal corresponding to each proton (see Supporting Information). ^1^H-NMR (400 MHz, DMSO-d_6_) *δ* 9.65 (s, 1H), 9.57 (s, 1H), 7.52 (s, 1H), 7.46 (s, 1H), 7.25–7.21 (m, 1H), 7.18–7.14 (m, 1H), 6.81–6.77 (m, 2H), 6.73–6.71 (m, 2H), 3.95 (s, 2H), 3.70 (s, 2H), 3.35–3.31 (m, 2H), 3.16–3.12 (m, 2H), 1.55–1.49 (m, 2H), 1.45–1.38 (m, 2H), 1.34–1.30 (m, 2H), 1.28 (s, 9H), 1.22 (s, 9H), 1.11–1.06 (m, 2H), 0.91 (t, *J* = 7.3 Hz, 3H), and 0.73 (t, *J* = 7.3 Hz, 3H). ^13^C-NMR (101 MHz, DMSO-d_6_) *δ* 171.3, 171.2, 167.9, 167.7, 138.7, 138.4, 129.9, 129.7, 117.2, 116.4, 111.0, 113.9, 113.6, 52.0, 50.7, 50.6, 49.6, 47.7, 45.9, 30.2, 29.2, 29.0, 28.9, 20.1, 19.6, 14.3, and 13.9. MS (IE, 70 eV) *m*/*z*(%): 306 (5); 249 (0.5); 233 (2); 206( 14); 192 (8); 121 (100); and 93 (15). HRMS (ESI+): *m*/*z* calculated for C_17_H_26_N_2_O_3_: 306.1943 [M]^+^, found: 307.2006.

*N-(1-(tert-butylaminocarbonyl)-1-3-hydroxyphenylmethyl)-N-(4-methylphenyl)-phenylcarboxamide* (**7g**). This compound was obtained by method A. The crude product was purified by flash column chromatography (hexane/ EtOAc, 8/2) to render the desired product as a white solid, m.p. (207–209) °C, yield 83%. ^1^H-NMR (400 MHz, CDCl_3_) *δ* 7.23 (d, *J* = 7.4 Hz, 2H), 7.20–7.16 (m, 1H), 7.11–7.05 (m, 4H), 6.89 (d, *J* = 7.6 Hz, 2H), 6.83–6.79 (m, 3H), 6.69 (d, *J* = 7.7 Hz, 1H), 6.18 (s, 1H), 6.02 (s, 1H), 2.16 (s, 3H), and 1.33 (s, 9H). ^13^C-NMR (101 MHz, CDCl_3_) *δ* 171.1, 169.0, 156.8, 138.7, 136.9, 136.3, 136.0, 129.8, 129.5, 129.4, 129.0, 128.5, 127.6, 121.4, 117.4, 115.8, 67.6, 51.7, 28.6, and 21.0. MS (IE, 70 eV) *m*/*z*(%): 417 (0.1); 317 (28); 212 (18); 105 (100); 91 (4); and 77 (16). HRMS (ESI+): *m*/*z* calculated for C_26_H_28_N_2_O_3_: 416.2100 [M]^+^, found: 417.2162

General procedure for the coupling of Ugi products and the furoxan derivative (**8a**–**8g**). The corresponding Ugi product (**7a**–**7g**) (0.2 mmol), the furoxan derivative **2** (0.2 mmol), and 1 mL of 2-MeTHF were placed in a round-bottom flask. Aqueous NaOH solution was added in four parts, every hour. Each time 20 µL was added, containing 0.3 eq of NaOH. The reaction mixture was then left stirring at room temperature for a total of 24 h. Subsequently, the reaction mixture was diluted with saturated NaHCO_3_ solution and extracted with EtOAc. The combined organic layers were dried with Na_2_SO_4_, filtered, and the solvent evaporated under reduced pressure. The residue was purified by flash column chromatography (SiO_2_ and hexane/EtOAc).

*N-[1-(tert-butylaminocarbonyl)-1-phenylmethyl]-N-(4-methylphenyl)-3-[3-(phenylsulfonyl)-[1,2,5]oxadiazol-4-yl N2-oxide]oxyphenylcarboxamide* (Ugi-Fx (**8a**)). This compound was obtained by the general procedure. The crude product was purified by flash column chromatography (hexane/ EtOAc, 7/3) to render the desired product as a white solid, m.p. (170–171) °C, yield 49%. ^1^H-NMR (400 MHz, CDCl_3_) *δ* 8.06–8.04 (m, 2H), 7.82–7.78 (m, 1H), 7.68–7.64 (m, 2H), 7.29–7.13 (m, 9H), 6.88–6.79 (m, 3H), 6.06 (s, 1H), 5.67 (s, 1H), 2.14 (s, 3H), and 1.38 (s, 9H). ^13^C-NMR (101 MHz, CDCl_3_) *δ* 169.3, 168.4, 157.9, 151.8, 138.3, 138.0, 137.9, 137.3, 135.9, 134.8, 130.2, 129.9, 129.8, 129.3, 129.2, 128.6, 128.5, 128.4, 126.9, 120.7, 120.2, 110.6, 67.2, 51.7, 28.7, and 21.0. MS (IE, 70 eV) *m*/*z*(%): 568 (M^+^-NH-*t*-butyl, 0.1); 540 (40); 345 (71); 316 (33); 196 (54); 121 (100); 91 (39); 77 (22); and 57 (14). HRMS (ESI+): calculated for. C_34_H_32_N_4_O_7_SNa: 663.1889, found: 663.1891.

*N-[1-(tert-butylaminocarbonyl)-1-phenylmethyl]-N-(4-methylphenyl)-2-[3-(phenylsulfonyl)-[1,2,5]oxadiazol-4-yl N2-oxide]oxyphenylcarboxamide* (**8b**). This compound was obtained by the general procedure. The crude product was purified by flash column chromatography (hexane/ EtOAc, 8/2) to render the desired product as a white solid, m.p. (100–102) °C, yield 70%. ^1^H-NMR (400 MHz, CDCl_3_) *δ* 8.28 (d, *J* = 7.5 Hz, 2H), 7.81 (t, *J* = 7.5 Hz, 1H), 7.69 (t, *J* = 7.8 Hz, 2H), 7.35–7.24 (m, 5H), 7.20–7.19 (m, 3H), 7.13–7.08 (m, 3H), 6.79 (d, *J* = 8.2 Hz, 2H), 6.16 (s, 1H), 5.59 (s, 1H), 2.13 (s, 3H), and 1.37 (s, 9H). ^13^C-NMR (101 MHz, CDCl_3_) *δ* 168.4, 167.1, 158.5, 148.3, 137.9, 137.3, 137.1, 135.6, 134.4, 130.4, 130.0, 129.9, 129.8, 129.7, 129.6, 129.0, 128.9, 128.4, 128.2, 126.3, 120.5, 110.9, 66.2, 51.7, 28.7, and 21.0. MS (IE, 70 eV) *m*/*z*(%): 568 (M^+^-NHtbutyl, 0.6); 540 (12); 345 (62); 316 (2); 196(100); 141 (46); 91 (35); 77 (51); and 57 (15). HRMS (ESI+): *m*/*z* calculated for C_34_H_32_N_4_O_7_SNa: 663.1889 [M+Na]^+^, found 663.1913.

*N-[1-(tert-butylaminocarbonyl)-1-phenylmethyl]-N-(4-methylphenyl)-4-[3-(phenylsulfonyl)-[1,2,5]oxadiazol-4-yl N2-oxide]oxyphenylcarboxamide* (**8c**). This compound was obtained by the general procedure. The crude product was purified by flash column chromatography (hexane/EtOAc, 7/3) to render the desired product as a white solid, m.p. (83–85) °C, yield 32%. ^1^H-NMR (400 MHz, CDCl_3_) *δ* 8.04 (dd, *J* = 8.5, 1.2 Hz, 2H), 7.79 (t, *J* = 7.5 Hz, 1H), 7.64 (t, *J* = 7.9 Hz, 2H), 7.42 (d, *J* = 8.9 Hz, 2H), 7.28–7.26 (m, 5H), 7.10 (d, *J* = 8.9 Hz, 2H), 6.91–6.82 (m, 4H), 6.08 (s, 1H), 5.69 (s, 1H), 2.20 (s, 3H), and 1.39 (s, 9H). ^13^C-NMR (101 MHz, CDCl_3_) *δ* 169.7, 168.5, 157.6, 153.0, 138.4, 137.8, 137.3, 135.8, 134.9, 134.7, 130.6, 130.1, 129.9, 129.8, 129.2, 129.1, 129.0, 128.6, 128.5, 128.4, 118.8, 110.7, 67.3, 51.7, 28.7, and 21.0. MS (IE, 70 eV) *m*/*z*(%): 568 (M^+^-NH-*t*-butyl, 0.8); 540 (25); 345 (92); 316 (13); 196 (100); 104 (18); 91(39); 77 (36); and 57 (16). HRMS (ESI+): *m*/*z* calculated for C_34_H_32_N_4_O_7_SNa: 663.1889 [M+Na]^+^, found 663.1913.

*N-(1-(tert-butylaminocarbonyl)methyl)-N-(4-methylphenyl)-3-[3-(phenylsulfonyl)-[1,2,5]oxadiazol-4-yl N2-oxide]oxy phenylcarboxamide* (**8d**). This compound was obtained by the general procedure. The crude product was purified by flash column chromatography (hexane/ EtOAc, 6/4) to render the desired product as a white solid, m.p. (65–67) °C, yield 61%. ^1^H-NMR (400 MHz, acetone-d_6_) *δ* 8.08 (dd, *J* = 8.5, 1.2 Hz, 2H), 7.94 (t, *J* = 8.1 Hz, 1H), 7.79 (t, *J* = 7.9 Hz, 2H), 7.39–7.30 (m, 4H), 7.13 (d, *J* = 8.3 Hz, 2H), 7.05 (d, *J* = 8.2 Hz, 2H), 6.90 (bs, 1H), 4.41 (s, 2H), 2.24 (s, 3H), and 1.35 (s, 9H). ^13^C-NMR (101 MHz, acetone-d_6_) *δ* 168.4, 167.1, 158.5, 152.5, 141.6, 138.7, 138.9, 136.5, 136.0, 129.9, 129.5, 129.4, 128.7, 127.4, 126.6, 120.6, 120.1, 111.2, 53.8, 50.6, 28.1, and 20.0. MS (IE, 70 eV) *m*/*z*(%): 564 (2); 492 (0.7); 345 (20); 240 (100); 146 (100); 91 (5); 77 (10); and 57 (11). HRMS (ESI+): *m*/*z* calculated for. C_28_H_28_N_4_O_7_S: 564.1679 [M]^+^, found: 565.1749.

*N-(1-(tert-butylaminocarbonyl)-1-phenylmethyl)-N-(butyl)-3-[3-(phenylsulfonyl)-[1,2,5]oxadiazol-4-yl N2-oxide]oxyphenylcarboxamide* (**8e**). This compound was obtained by the general procedure. The crude product was purified by flash column chromatography (hexane/ EtOAc, 6/4) to render the desired product as an amorphous white solid, yield 42%. ^1^H-NMR (400 MHz, DMSO-d_6_) *δ* 8.08 (dd, *J* = 8.4; 1.1 Hz, 2H), 7.94 (t, *J* = 7.5 Hz, 1H), 7.79 (t, *J* = 7.9 Hz, 2H), 7.62–7.58 (m, 3H), 7.43–7.30 (m, 6H), 6.06 (bs, 1H), 5.27 (bs, 1H), 3.20–3.07 (m, 2H), 1.25 (s, 9H), 1.06–0.9 (m, 1H), and 0.67–0.36 (m, 6H). ^13^C-NMR (101 MHz, DMSO-d_6_) *δ* 176.3, 169.0, 164.9, 153.1, 139.5, 137.4, 136.7, 131.1, 130.5, 130.3, 129.6, 129.1, 128.9, 128.5, 124.7, 121.1, 118.2, 111.8, 65.3, 60.2, 45.3, 31.5, 28.7, 19.7, and 13.6. MS (IE, 70 eV) *m*/*z*(%): 606 (0.1); 490 (29); 345 (42); 282 (44); 261 (13); 121 (100); 77 (28); and 57 (19). HRMS (ESI+): *m*/*z* calculated for. C_31_H_34_N_4_O_7_: 606.2148 [M]^+^, found: 607.2239.

*N-(1-(tert-butylaminocarbonyl)methyl)-N-(butyl)-3-[3-(phenylsulfonyl)-[1,2,5]oxadiazol-4-yl N2-oxide]oxyphenylcarboxamide* (**8f**). This compound was obtained by the general procedure. The crude product was purified by flash column chromatography (hexane/ EtOAc, 6/4) to render the desired product as an amorphous white solid, yield 37%. Due to the tautomeric equilibrium, the signals of the compound in the ^1^H-NMR are duplicated at room temperature (298 K). When heating to 348 K, the phenomenon of coalescence is observed, and there is a signal corresponding to each proton. ^1^H-NMR (400 MHz, DMSO-d_6_) *δ* 8.08–8.04 (m, 4H), 7.95–7.91 (m, 2H), 7.80–7.76 (m, 4H), 7.59–7.46 (m, 6H), 7.35–7.33 (m, 2H), 3.99 (s, 2H), 3.72 (s, 2H), 3.37–3.34 (m 2H), 3.15–3.12 (m, 2H), 1.57–1.49 (m, 2H), 1.45–1.40 (m, 2H), 1.28 (s, 9H), 1.19 (s, 9H), 1.10–1.03 (m, 2H), 0.92 (t, *J* = 7.3 Hz, 3H), and 0.71 (t, *J* = 7.3 Hz, 3H). ^13^C-NMR (101 MHz, DMSO-d_6_) *δ* 169.9, 169.8, 167.7, 167.4, 159.0, 158.9, 152.7, 152.6, 139.2, 139.0, 137.4, 136.7, 130.9, 130.7, 130.5, 129.1, 129.0, 128.7, 125.3, 125.1, 121.0, 118.5, 112.2, 111.7, 52.1, 50.7, 50.6, 49.9, 47.9, 46.3, 30.2, 29.2, 29.0, 28.7, 20.1, 19.6, 14.3, and 13.8. MS (IE, 70 eV) *m*/*z*(%): 530 (5); 458 (4); 416 (29); 345 (100); 290 (17); 185 (70); 114 (16); 77 (45); and 57 (46). HRMS (ESI+): *m*/*z* calculated for. C_25_H_30_N_4_O_7_S: 530.1835 [M]^+^, found: 531.1925.

*N-(1-(tert-butylaminocarbonyl)-1-3-[3-(phenylsulfonyl)-[1,2,5]oxadiazol-4-yl N2-oxide]oxy phenylmethyl)-N-(4-methylphenyl)-phenylcarboxamide* (**8g**). This compound was obtained by the general procedure. The crude product was purified by flash column chromatography (hexane/ EtOAc, 7/3) to render the desired product as a white solid, m.p. (69–71) °C, yield 58%. ^1^H-NMR (400 MHz, CDCl_3_) *δ* 8.15–8.09 (m, 3H), 7.83–7.77 (m, 1H), 7.69–7.62 (m, 2H), 7.52–7.48 (m, 2H), 7.37–7.32 (m, 4H), 7.25–7.14 (m, 4H), 6.88–6.84 (m, 2H), 6.11 (s, 1H), 6.09 (s, 1H), 2.17 (s, 3H), and 1.40 (s, 9H). ^13^C-NMR (101 MHz, CDCl_3_) *δ* 171.4, 168.0, 158.3, 152.4, 138.3, 137.8, 137.6, 137.3, 135.9, 135.7, 133.7, 130.2, 129.9, 129.8, 129.7, 129.6, 129.4, 128.7, 128.6, 128.5, 127.7, 121.8, 119.7, 66.5, 51.9, 28.7, and 20.9. MS (IE, 70 eV) *m*/*z*(%): 568 (M^+^-NHtbutyl, 0.1); 540 (4); 294 (0.2); 105 (100); 91 (2); 77 (14); and 57 (3). HRMS (FAB): *m*/*z* [M+Na]^+^ calculated for C_34_H_32_N_4_O_7_SNa: 663.1889, found: 663.1893.

General Experimental Procedure for GBB-3C Reaction (**10**, **12a**–**e**). Monowave-50-Assisted Synthesis. A glass tube was charged sequentially with EtOH (2.0 mL/mmol), 5-HMF (1.0 equiv), amidine (1.0 equiv), isocyanide (1.1 equiv), and TFA (20 mol%). The test tube was then sealed with a Teflon septum and heated for 2 h at 60 °C (Method A) or 20 min at 120 °C (Method B). Different purification procedures were carried out for each corresponding GBB derivative. 

*N-tert-Butyl-2-(3-hydroxyphenyl)imidazo[1,2-a]pyridin-3-amine* (**10**). This compound was obtained by method A or B. The corresponding product precipitated in the crude reaction mixture after 24 h in the refrigerator. The solid was filtered, washed with cold ethanol, and dried to render the desired product as a white crystalline solid, m.p. (236–238) °C, yield 62% (method A) and 70% (method B). ^1^H-NMR (400 MHz, MeOD) *δ* 8.38 (dt, *J* = 6.9, 1.2 Hz, 1H), 7.48–7.32 (m, 3H), 7.26–7.20 (m, 2H), 6.90 (td, *J* = 6.8, 1.0 Hz, 1H), 6.77 (ddd, *J* = 8.1, 2.5, 0.9 Hz, 1H), and 1.00 (s, 9H). ^13^C-NMR (101 MHz, MeOD) *δ* 157.1, 141.7, 138.7, 136.1, 128.9, 124.8, 124.2, 123.9, 119.6, 115.6, 115.0, 114.2, 111.5, 55.6, and 29.2. MS (EI, 70 eV) *m*/*z* (%): 281(45), 224(78), 197(100), and 78(97). HRMS (ESI+): *m*/*z* calculated for C_17_H_19_N_3_O 281.1528 [M]^+^, found 282.1592 [M+1]^+^. 

*4-(3-(3-(tert-butylamino)imidazo[1,2-a]pyridin-2-yl)phenoxy)-3-(phenylsulfonyl)-1,2,5-oxadiazole 2-oxide* (**12a**). This compound was obtained by method A. The corresponding product precipitated in the crude reaction mixture after 24 h in the refrigerator. The solid was filtered, washed with cold ethanol, and dried to render the desired product as a yellow solid, m.p. (77–79) °C, yield 54% (method A). When this compound was synthesized by method B 12a did not precipitate in the crude reaction mixture and was purified by flash column chromatography (hexane/ EtOAc, 8/2) to render the desired product in 75% yield. ^1^H-NMR (400 MHz, acetone-d_6_) *δ* 8.45 (d, *J* = 6.9 Hz, 1H), 8.29 (dd, *J* = 2.4, 1.5 Hz, 1H), 8.23 (dt, *J* = 8.0, 1.2 Hz, 1H), 8.17–8.09 (m, 2H), 7.91 (tt, *J* = 7.5, 1.2, Hz, 1H), 7.82–7.73 (m, 2H), 7.54 (t, *J* = 8.0 Hz, 1H), 7.48 (d, *J* = 9.0 Hz, 1H), 7.34 (ddd, *J* = 8.2, 2.6, 0.9 Hz, 1H), 7.23 (ddd, *J* = 9.0, 6.6, 1.2 Hz, 1H), 6.90 (td, *J* = 6.8, 1.0 Hz, 1H), 4.15 (s, NH), and 1.11 (s, 9H). ^13^C-NMR (101 MHz, acetone-d_6_) *δ* 159.9, 154.0, 142.6, 139.0, 138.9, 138.0, 136.9, 130.8, 130.5, 129.6, 126.9, 125.3, 125.1, 119.9, 119.3, 117.9, 112.3, 57.0, and 30.6. MS (EI, 70 eV) *m*/*z* (%): 505(7), 449(13), 281(17), 250(9), 224(40), 197(45), and 78(100). HRMS (ESI+): *m*/*z* calculated for C_25_H_23_N_5_O_5_S 505.1420 [M]^+^, found 506.1497 [M+1]^+^. 

*4-(2-(3-(tert-butylamino)imidazo[1,2-a]pyridin-2-yl)phenoxy)-3-(phenylsulfonyl)-1,2,5-oxadiazole 2-oxide* (**12b**). This compound was obtained by method A. The crude reaction was concentrated under reduced pressure to eliminate ethanol, and the product was purified by flash column chromatography (hexane/ EtOAc, 8/2) to render a white solid, m.p. (139–141) °C, yield 32%. ^1^H-NMR (400 MHz, acetone-d_6_) *δ* 8.59 (d, *J* = 6.9 Hz, 1H), 8.09 (dd, *J* = 8.5, 1.2 Hz, 2H), 7.98–7.93 (m, 1H), 7.83–7.77 (m, 1H), 7.68–7.59 (m, 3H), 7.57–7.46 (m, 2H), 7.36–7.31 (m, 1H), 7.20 (ddd, *J* = 9.0, 6.6, 1.3 Hz, 1H), 6.96 (td, *J* = 6.8, 1.1 Hz, 1H), 4.16 (s, 1H), and 1.03 (s, 9H). ^13^C-NMR (101 MHz, acetone-d_6_) *δ* 160.2, 150.5, 138.6, 136.8, 133.4, 130.6, 129.9, 129.6, 128.0, 124.9, 122.4, 118.1, 112.3, 56.7, and 30.2. MS (EI, 70 eV) *m*/*z* (%): 505(0.9), 432(13), 281(14), 250(14), 224(44), 197(35), and 78(100). HRMS (ESI+): *m*/*z* calculated for C_25_H_23_N_5_O_5_S 505.1420 [M]^+^, found 506.1498 [M+1]^+^. 

*4-(4-(3-(tert-butylamino)imidazo[1,2-a]pyridin-2-yl)phenoxy)-3-(phenylsulfonyl)-1,2,5-oxadiazole 2-oxide* (**12c**). This compound was obtained by method A. The corresponding product precipitated in the crude reaction mixture after 24 h in the refrigerator. The solid was filtered, washed with cold ethanol, and dried to render the desired product as a white solid, m.p. (160–161) °C, yield 64%. ^1^H-NMR (400 MHz, acetone-d_6_) *δ* 8.44 (d, *J* = 6.9 Hz, 1H), 8.39–8.34 (m, 2H), 8.19–8.09 (m, 2H), 7.96–7.90 (m, 1H), 7.83–7.74 (m, 2H), 7.53–7.42 (m, 3H), 7.21 (ddd, *J* = 9.0, 6.6, 1.2 Hz, 1H), 6.88 (td, *J* = 6.8, 1.0 Hz, 1H), 4.16 (s, NH), and 1.13 (s, 9H). ^13^C-NMR (101 MHz, acetone-d_6_) *δ* 159.7, 152.9, 142.7, 138.9, 138.7, 136.8, 135.4, 130.7, 130.3, 129.6, 124.9, 124.8, 120.1, 117.9, 112.2, 111.9, 56.9, and 30.7. MS (EI, 70 eV) *m*/*z* (%): 505(5), 448(9), 421 (8), 281(12), 250(9), 224(68), 197(69), and 78(100). HRMS (ESI+): *m*/*z* calculated for C_25_H_23_N_5_O_5_S 505.1420 [M]^+^, found 506.1492 [M+1]^+^.

*4-(3-(3-(tert-butylamino)imidazo[1,2-a]pyrazin-2-yl)phenoxy)-3-(phenylsulfonyl)-1,2,5-oxadiazole 2-oxide* (**12d**). This compound was obtained by method A. The crude reaction was concentrated under reduced pressure to eliminate ethanol, and the product was purified by flash column chromatography in CHCl_3_ as the mobile phase to render a white solid, m.p. (156–157) °C, yield 50%; ^1^H-NMR (400 MHz, acetone-d_6_) *δ* 8.89 (d, *J* = 1.4 Hz, 1H), 8.43 (dd, *J* = 4.7, 1.4 Hz, 1H), 8.34 (dd, *J* = 2.4, 1.5 Hz, 1H), 8.29–8.26 (m, 1H), 8.16 (dd, *J* = 8.5, 1.2 Hz, 2H), 7.95–7.90 (m, 1H), 7.87 (d, *J* = 4.6 Hz, 1H), 7.82–7.77 (m, 2H), 7.61 (t, *J* = 8.0 Hz, 1H), 7.42 (ddd, *J* = 8.2, 2.6, 0.9 Hz, 1H), 4.33 (s, 1H), and 1.14 (s, 9H). ^13^C-NMR (101 MHz, acetone-d_6_) *δ* 159.8, 154.1, 144.0, 140.6, 138.9, 138.2, 138.1, 136.9, 130.8, 130.7, 129.6, 127.0, 120.1, 119.8, 117.9, 112.1, 57.5, and 30.6. MS (EI, 70 eV) *m*/*z* (%): 506(18), 450(61), 434(24), 282(37), 241(34), 226(85), 198(39), 181(23), 79(98), 57(100), and 41(47). HRMS (ESI+): *m*/*z* calculated for C_24_H_22_N_6_O_5_S 506.1372 [M]^+^, found 507.1449 [M+1]^+^.

*4-(3-(5-(tert-butylamino)imidazo[2,1-b]thiazol-6-yl)phenoxy)-3-(phenylsulfonyl)-1,2,5-oxadiazole 2-oxide* (**12e**). This compound was obtained by method A. The crude reaction was concentrated under reduced pressure to eliminate ethanol, and the product was purified by flash column chromatography in CHCl_3_ as the mobile phase to render a white solid, m.p. (76–77) °C, yield 38%; ^1^H-NMR (400 MHz, acetone-d_6_) *δ* 8.25 (dd, *J* = 2.4, 1.5 Hz, 1H), 8.19 –8.16 (m, 1H), 8.13 (dt, *J* = 3.3, 1.4 Hz, 2H), 7.93–7.88 (m, 1H), 7.79–7.74 (m, 2H), 7.72 (d, *J* = 4.6 Hz, 1H), 7.47 (t, *J* = 8.0 Hz, 1H), 7.24 (ddd, *J* = 8.2, 2.6, 0.9 Hz, 1H), 7.09 (d, *J* = 4.6 Hz, 1H), 4.02 (s, NH), and 1.13 (s, 9H). ^13^C-NMR (101 MHz, acetone-d_6_) *δ* 159.8, 156.8, 153.9, 139.1, 139.0, 136.8, 130.8, 130.3, 129.6, 127.6, 125.7, 119.7, 118.7, 118.1, 112.6, 112.1, 56.5, and 30.4. MS (EI, 70 eV) *m*/*z* (%): 511 (9), 439(26), 411(21), 287(23), 228(54), 203(76), 141(25), 85(40), 77(100), and 57(49). HRMS (ESI+): *m*/*z* calculated for C_23_H_21_N_5_O_5_S_2_ 511.0984 [M]^+^, found 512.1059 [M+1]^+^.

### 3.2. Biology

#### 3.2.1. Materials

The cell culture supplies were from Gibco (Gaithersburg, MD, USA), Capricorn Scientific (Ebsdorfergrund, Germany), and Biological Industries (Beit Haemek, Israel). The SRB, DMSO, and cisplatin were from Sigma-Aldrich (St. Louis, MO, USA). The bladder cancer cells T24 (ATCC HBT-4) were purchased from the Cell Repository ABAC (Asociación Banco Argentino de Células). The prostate carcinoma cells LNCaP were purchased from ATCC (CRL-174). The immortalized human keratinocytes HaCaT (BCRJ batch number 001071) were kindly provided by Dr. Jimena Hochmann. 

#### 3.2.2. Cell Culture

The T24 cells were grown in McCoy’s 5A (Biological Industries) supplemented with 10% FBS (Capricorn Scientific). The cells were passaged twice per week, and the culture medium was changed with the same frequency. The LNCaP cells were maintained in RPMI media (Biological Industries) supplemented with 10% FBS (Capricorn Scientific) and 10% of its own conditioned medium (complete media). The cells were passaged once per week, and the medium was changed with the same frequency. The HaCaT cells (immortalized human keratinocytes) were cultured in Dulbecco’s modified Eagle medium (Biological Industries) supplemented with 10% FBS (Capricorn Scientific). The cells were passaged twice per week, and the medium was changed with the same frequency. The cell cultures were maintained under a humidified 5% CO_2_ atmosphere at 37 °C. 

#### 3.2.3. Antiproliferative Activity

For viability assays, cells were treated with a 0.25% Trypsin–0.05% EDTA solution and plated in 96-well plates in complete medium the day before the experiment. The cells were seeded in a 96-well plate at densities of 8 × 10^3^ cells per well for T24 cells, 12.5 × 10^3^ cells per well for LNCaP cells, and 10 × 10^3^ cells per well for HaCaT cells. The cultures were allowed to attach for 24 h in a humidified 5% CO_2_ atmosphere at 37 °C. Then, the cells were treated with increasing concentrations of the compounds (0.4–100.0 µM) prepared in complete medium using stock solutions in DMSO and were kept for 48 h in a CO_2_ incubator. Wells with only media or 0.5% DMSO were added as controls. The antiproliferative activity of the compounds was assayed using the SRB method in at least three independent experiments [33].

#### 3.2.4. Nitric-Oxide-Releasing Activity

The NO-releasing ability of the synthesized compounds was determined in T24 and LNCaP cell cultures as we previously described [32]. Cells were seeded in a 96-well plate at densities of 8 × 10^3^ and 12.5 × 10^3^ cells per well, respectively, and were allowed to attach for 24 h in a humidified 5% CO_2_ atmosphere at 37 °C. Afterward, the culture milieu was removed, and the solubilized compounds in DMSO were added at the final concentration of 100 µM diluted in fresh culture medium in triplicate. Controls with medium and 0.5% DMSO were included in each experiment. The cells were further incubated for 3 h at 37 °C and 5% CO_2_, and the NO production was measured as the nitrite content in the culture medium by the Griess reaction. To develop this assay, 50 µL of culture medium was transferred to a new multi-well plate. At the same time, a standard curve with NaNO_2_ was performed at serial dilutions between 0 and 100 µM in 50 µL of culture medium. Then, 50 µL of 1% sulfanilamide solution in 5% phosphoric acid was added to each well and incubated for 10 min while protected from light. Then, 50 µL of 0.1% N-1-naphthylethylendiamine dihydrochloride in water was added, and the plates were incubated for another 10 min in the dark. The absorbance was measured at 540 nm using a microplate spectrophotometer (Varioskan Flash Microplate spectrophotometer; Thermo Fisher, Vantaa, Finland). The NO concentration for each condition was determined in at least three independent experiments.

#### 3.2.5. Antiproliferative Activity with Hemoglobin

To investigate the contribution of ⋅NO to the antiproliferative activity of the studied compounds, we tested their effect on T24 bladder cancer cell growth in the absence and presence of Hb. Cells were seeded into a 96-well plate at 8 × 10^3^ cells per well and were allowed to attach for 24 h. The cultures were pretreated with Hb (0 µM or 50 µM) for 1 h and then treated with 10 µM of the selected compounds for 48 h. Then, the antiproliferative activity was assessed by the SRB assay in at least three independent experiments. A statistical analysis was carried out using a two-way analysis of variance (ANOVA).

## 4. Conclusions

In conclusion, we report the synthesis of 12 compounds derived from furoxan and its precursors via the multicomponent reactions of Ugi and GBB under greener conditions. These MCRs were performed under mild temperature conditions and short reaction times using the Monowave 50 reactor and EtOH as a green solvent with yields of 25–96%. The minilibrary of compounds obtained was assayed against two human cancer cell lines, with most of them displaying the ability to inhibit cell growth. Higher activity was observed in the T24 bladder cancer line than in the LNCaP prostate cancer line. Our studies showed that every compound was able to release ·NO in the presence of T24 and LNCaP cells. The antiproliferative activity of the compounds declined in the presence of an ·NO scavenger (hemoglobin), suggesting that ·NO release is partially involved in their anticancer mechanism. Compounds Ugi-Fx (**8a**) and **12e** exhibited the most potent activity (GI_50_ of 730 and 830 nM, respectively), being more active than the reference drug cisplatin on the T24 cell line and similar to it on the LNCaP cell line. Both compounds proved to have a ten-fold higher selectivity for T24 cancer cells than for non-cancer cells HaCaT, surpassing cisplatin and also the furoxan derivative **2**. Our results demonstrate that coupling the furoxan system to structures derived from multicomponent reactions can enhance antiproliferative activity and selectivity, particularly in the T24 bladder cancer line. Concerning the LNCaP cell line, it is worth mentioning that imidazo[1,2-a]pyrazine **12d** stands out, with an SI value 10 times higher than the reference drug cisplatin, and was, hence, identified as starting point in the design of new GBB furoxan derivatives with improved selectivity. Additional studies are being performed to further explore the anticancer mechanism of compounds **8a**, **12d**, and **12e**. The results will be reported in due course.

## Figures and Tables

**Table 1:**
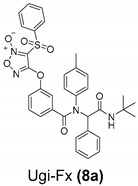
Structure of ⋅NO-donor Ugi derivative **8a** with antiproliferative activity against six cancer cells lines and its selectivity index (SI) in comparison to the non-cancer HaCaT human cells [9].

	A549 (Lung)	HBL-100 (Breast)	HeLa (Cervix)	SW1573 (Lung)	T-47D (Breast)	WiDr (Colon)
**GI_50_ (** **µM)**	2.3 (±0.5)	0.23 (±0.02)	1.8 (±0.3)	0.021 (±0.013)	1.9 (±0.3)	3.0 (±0.6)
**SI**	4.3	43	5.5	471.4	5.2	3.3

**Table 2:** Antiproliferative activity (GI_50_, μM) ^a^ against bladder (T24) and prostate (LNCaP) human cancer cells of compounds obtained via Ugi and GBB reactions.

Compound	Cell Line		Compound	Cell Line	
	T24	LNCaP		T24	LNCaP
Furoxan reference compounds					
**3**	8.60 (±1.68)	32.46 (±9.96)	**11a**	7.08 (±1.70)	24.52 (±5.20)
Ugi products			GBB products		
**7a**	>100	>100	**10**	>100	>100
Ugi-Fx (**8a**)	0.73 (±0.12)	20.2 (±4.66)	**12a**	1.76 (±0.34)	24.7 (±1.83)
**8b**	1.83 (±0.59)	13.3 (±5.60)	**12b**	0.85 (±0.25)	7.70 (±2.13)
**8c**	2.18 (±0.63)	18.8 (±1.62)	**12c**	2.99 (±0.98)	24.2 (±3.40)
**8d**	2.10 (±0.45)	21.79 (±8.39)	**12d**	2.72 (±0.41)	8.13 (±1.55)
**8e**	1.53 (±0.29)	21.82 (±6.09)	**12e**	0.83 (±0.14)	16.53 (±4.99)
**8f**	2.86 (±0.46)	29.87 (±6.96)	Positive control		
**8g**	1.70 (±0.46)	36.7 (±11.8)	Cisplatin	3.28 (±1.20)	20.3 (±4.20)

^a^ Mean (±standard error of the mean).

**Table 3:** Cytotoxicity of Ugi and GBB derivatives against HaCaT cells, along with selectivity indices.

Compound	HaCaT GI_50_ (µM) ^a^	*SI* ^b^	
		T24	LNCaP
**2**	1.47 (±1.11) ^c^	1.1^c^	ND
**3**	14.40 (±1.92)	1.7	0.4
**11a**	7.83 (±1.43)	1.1	0.3
Ugi-Fx (**8a**)	10.58 (±3.08)	14.5	0.5
**8b**	3.78 (±1.15)	2.1	0.3
**8c**	4.62 (±0.99)	2.1	0.2
**8d**	9.99 (±2.70)	4.8	0.5
**8e**	11.78 (±2.57)	4.5	0.5
**8f**	11.85 (±2.73)	7.7	0.4
**8g**	4.99 (±1.19)	2.9	0.1
**12a**	7.44 (±2.13)	4.2	0.3
**12b**	3.11 (±0.94)	3.7	0.4
**12c**	5.22 (±1.20)	1.7	0.2
**12d**	9.84 (±2.06)	3.6	1.2
**12e**	9.01 (±2.06)	10.8	0.2
Cisplatin	2.51 (±1.19)	0.8	0.1

^a^ Mean (±standard error of the mean). ^b^ Selectivity index: (GI_50_ (HaCaT))/(GI_50_ (cancer cell line)). ^c^ Data from [33]. ND: Not determined.

## Data Availability

Not applicable.

## Supplementary Materials

The following supporting information can be downloaded at: https://www.mdpi.com/article/10.3390/molecules27061756/s1, Table S1: Levels of NO produced by the compounds during incubation in physiological solution in the presence of L-cysteine, Figures of 1H, 13C-NMR spectra of synthesized compounds and 2D NOESY NMR for compounds **10** and **12a**.

## Sample Availability

Samples of the compounds are available from the authors.
