# Supplementary material for: Greener Synthesis of Antiproliferative Furoxans via Multicomponent Reactions
Source: Molecules. 2022 Mar 8;27(6):1756. doi: 10.3390/molecules27061756 (PMC8955377; doi:10.3390/molecules27061756)
Supplement: Supplementary file 1 [file molecules-27-01756-s001.zip › molecules-1558399-supplementary.pdf]

Supporting Information

Article

Greener synthesis of antiproliferative furoxans via multicomponent reactions

Mariana Ingold, Victoria de la Sovera, Rosina Dapueto, Paola Hernández, Williams Porcal, Gloria V. López.

Table of Contents

NO release in physiological solution **2**

NMR data of synthesized compounds **4**

NO release in physiological solution

NO produced by the compounds during incubation in physiological solution in the presence of L-cysteine was determined. The compounds were solubilized in DMSO and then diluted at 100 µM in a mixture 50 mM pH 7.4 PBS solution/MeOH (1% DMSO) 50/50 v/v. Subsequently they were incubated in the presence of L-cysteine at a 0.5 mM concentration (a 5-fold excess compared to the NO-donor derivative). After 1 h and 3 h at 37 °C, the presence of nitrite in the sample was determined using the Griess assay. To develop this assay, 50 µL of the mixture was transferred to a new multi-well plate. At the same time, a standard curve with NaNO2 was performed at serial dilutions between 0 and 100 µM in 50 µL of the PBS/MeOH mixture. After that, 50 µL of 1% sulfanilamide solution in 5% phosphoric acid was added to each well and incubated for 10 min protected from light. Then 50 µL of 0.1% N-1-naphthylethylendiamine di-hydrochloride in water was added and the plates were incubated for another 10 min in the dark. The absorbance was measured at 540 nm using a microplate spectrophotometer (Varioskan Flash Microplate spectrophotometer; Thermo Fisher, Vantaa, Finland).

Table S1. Levels of NO produced by the compounds during incubation in physiological solution in the presence of L-cysteine.

**Compound**	**% NO_2– (mol/mol)** [a]		
	**without L-Cys, 1h incubation**	**with L-Cys, 1h incubation** [a, b]	**with L-Cys, 3 h incubation** [a, c]
**Furoxan reference compounds**			
**2**	0.63 (± 0.80)	37.71 (± 1.16)	39.93 (± 2.19)
**3**	ND	31.81 (± 1.21)	31.83 (± 1.15)
**11a**	ND	19.84 (± 1.35)	20.02 (± 0.95)
**7a**	ND	0.71 (± 0.39)	2.56 (± 1.77)
**10**	ND	0.83 (± 0.56)	2.76 (± 1.45)0
**Ugi-Furoxan products**			
**Ugi-Fx (8a)**	ND	38.45 (±1.14)	43.88 (± 1.96)
**8b**	ND	37.07 (±1.59)	44.23 (± 2.81)
**8c**	ND	33.40 (±2.32)	36.79 (± 3.26)
**8d**	ND	27.89 (±1.84)	39.93 (± 2.31)
**8e**	ND	34.62 (±1.86)	38.43 (± 1.57)
**8f**	ND	32.65 (±1.00)	39.79 (± 2.68)
**8g**	ND	35.92 (±1.45)	40.51 (± 3.08)
**GBB-Furoxan products**			
**12a**	ND	17.22 (±1.18)	22.025 (± 1.26)
**12b**	ND	30.38 (±1.66)	35.97 (± 1.55)
**12c**	ND	19.02 (±1.12)	24.10 (± 2.43)

**12d**	ND	16.59 (±1.36)	19.07 (± 1.87)
**12e**	ND	13.50 (±1.14)	16.31 (± 1.19)
**SNAP**	ND	18.18 (± 1.30)	20.11 (± 2.21)

[a] Mean (± standard error of the mean).

[b] Determined by Griess reaction, after incubation for 1 h at 37°C in pH 7.4 buffered water-methanol mixture, in the presence of a 1:5 molar excess of L-cysteine.

[c] Determined by Griess reaction, after incubation for 3 h at 37°C in pH 7.4 buffered water-methanol mixture, in the presence of a 1:5 molar excess of L-cysteine.

^{1}H and ^{13}C-NMR spectra

Ugi reaction derivatives

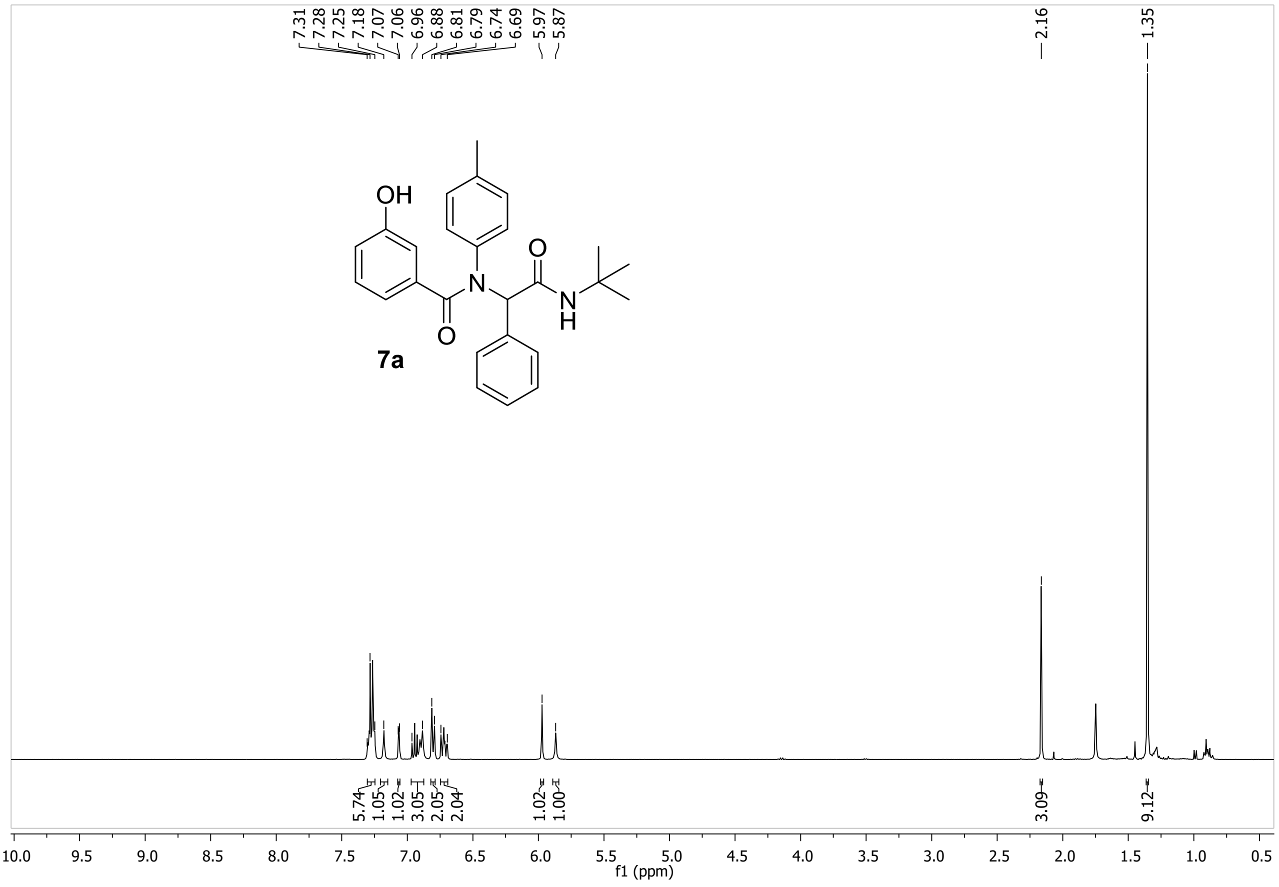

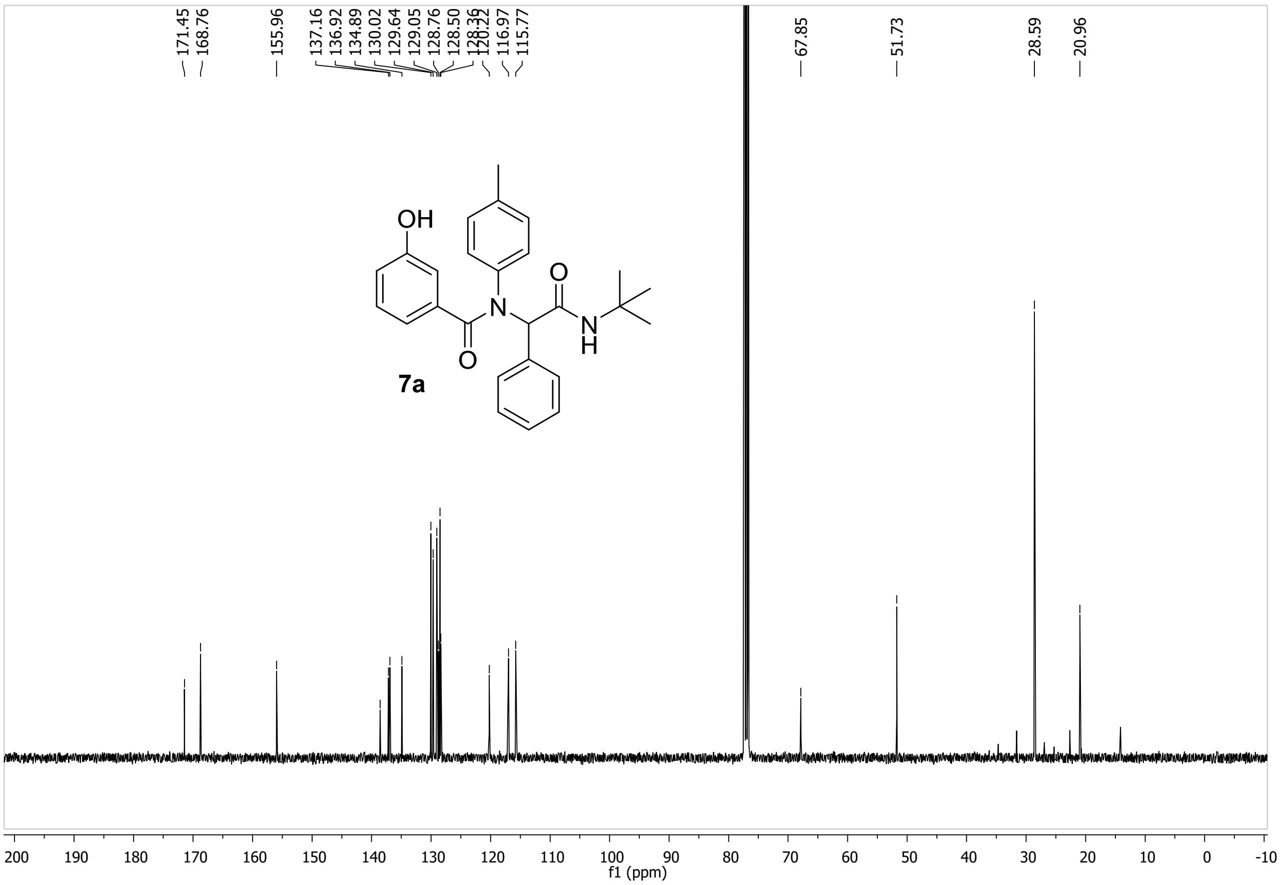
171.45
168.76
155.96
137.16
136.92
134.89
130.02
129.64
129.05
128.76
128.50
128.36
120.22
116.97
115.77
67.85
51.73
28.59
20.96
OH
O
N
N
H
O
7a
200 190 180 170 160 150 140 130 120 110 100 90 80 70 60 50 40 30 20 10 0 -10
f1 (ppm)

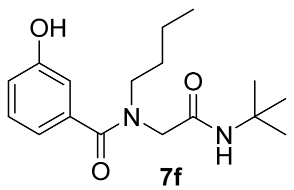

7f

Due to the tautomeric equilibrium, the signals of the compound **7f** in the ^{1}H-NMR are duplicated at room temperature (298 K), when heating to 348 K the phenomenon of coalescence is observed and there is a signal corresponding to each proton.

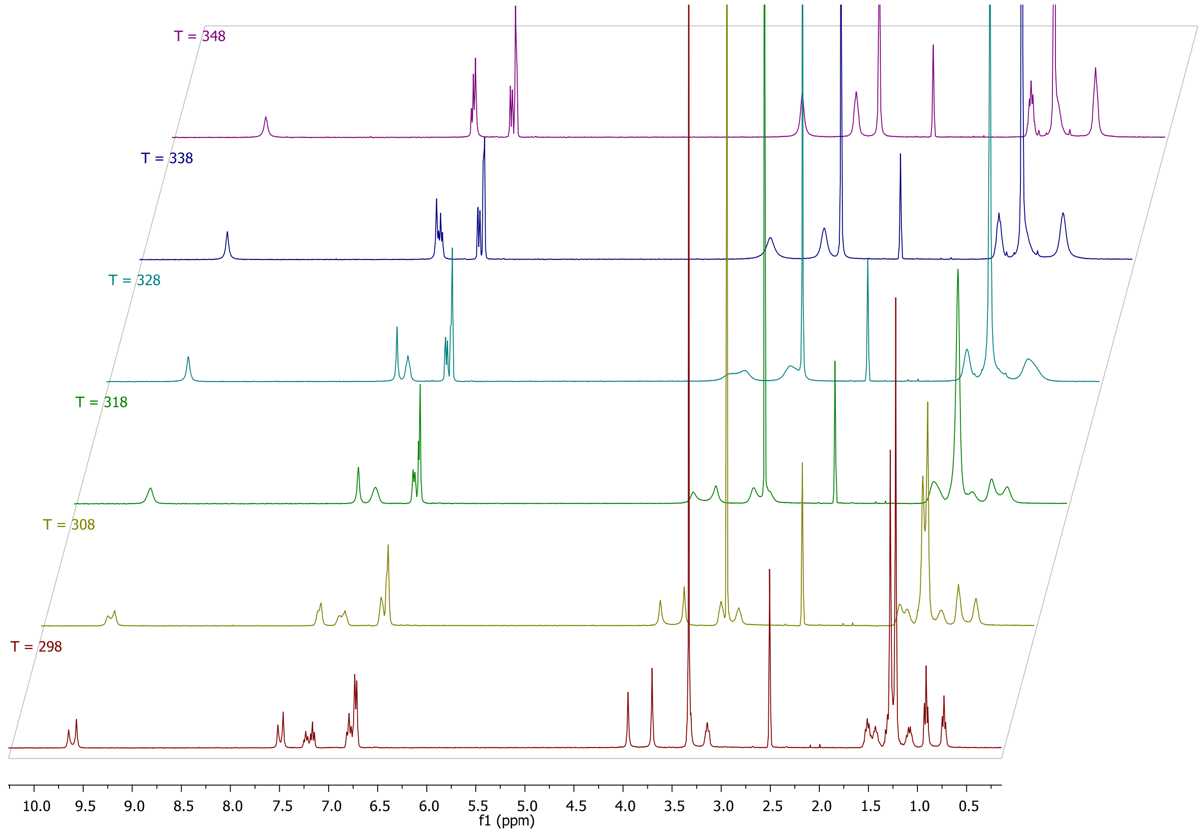

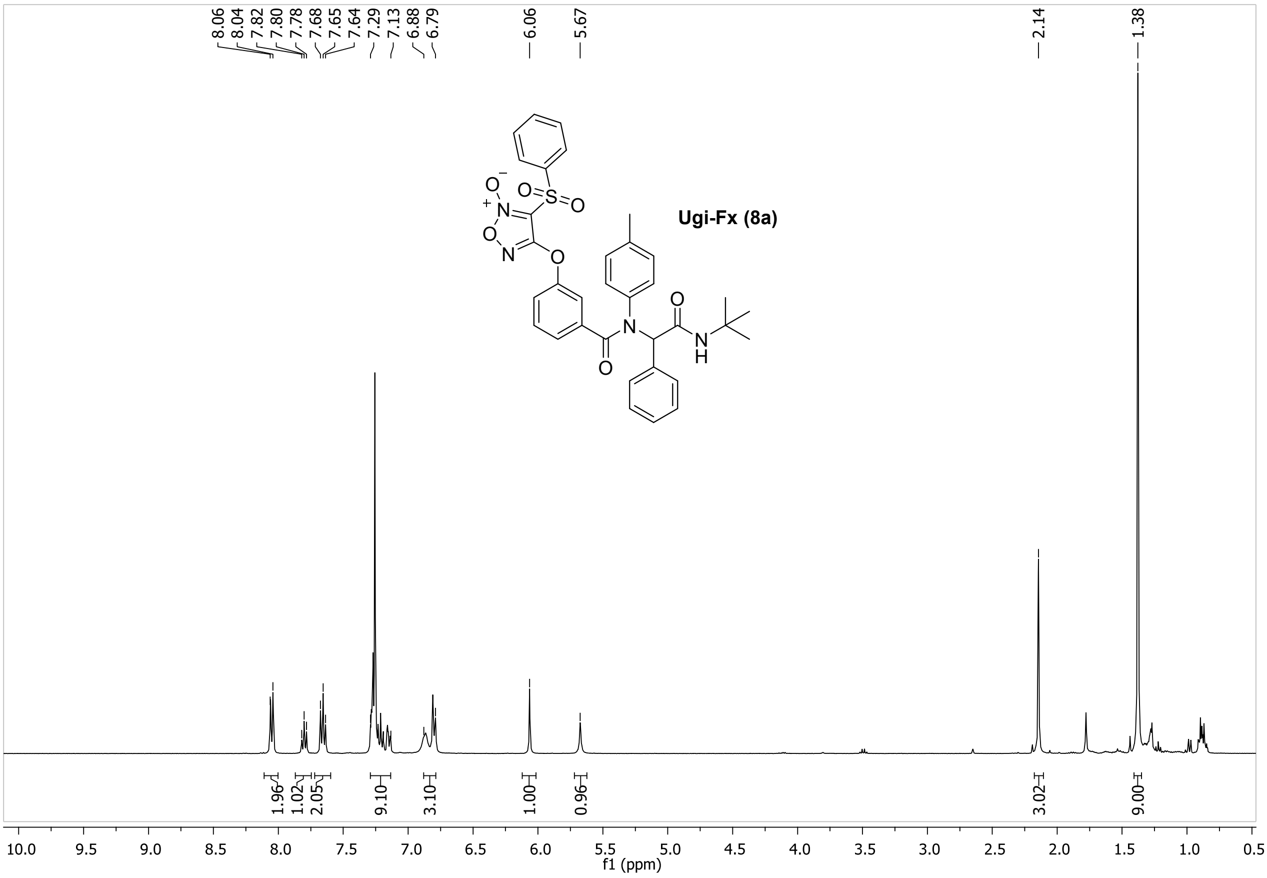
8.06
8.04
7.82
7.80
7.78
7.68
7.65
7.64
7.29
7.13
6.88
6.79
6.06
5.67
2.14
1.38
Ugi-Fx (8a)
1.96
1.02
2.05
9.10
3.10
1.00
0.96
3.02
9.00
f1 (ppm)

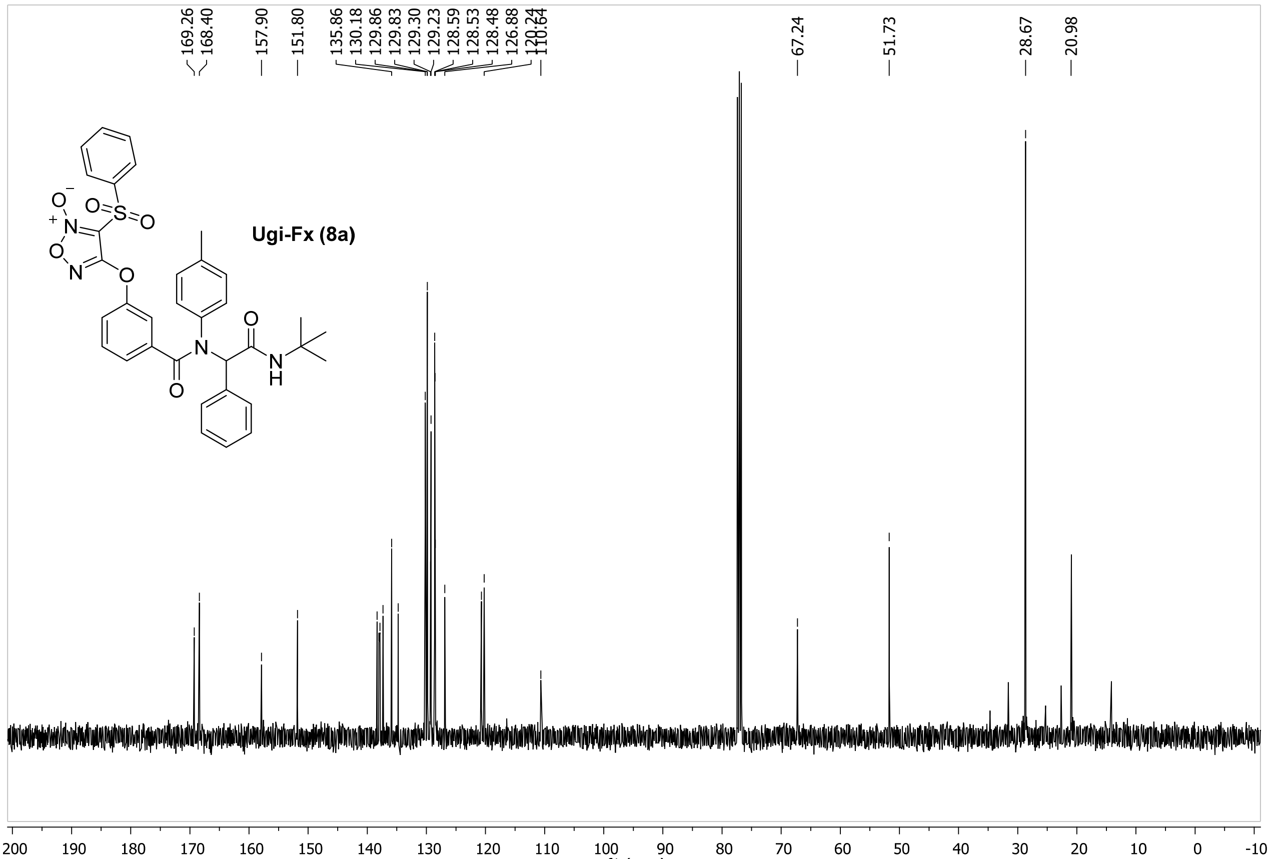
169.26
168.40
157.90
151.80
135.86
130.18
129.86
129.83
129.30
129.23
128.59
128.53
128.48
126.88
120.24
110.64
67.24
51.73
28.67
20.98
Ugi-Fx (8a)
f1 (ppm)

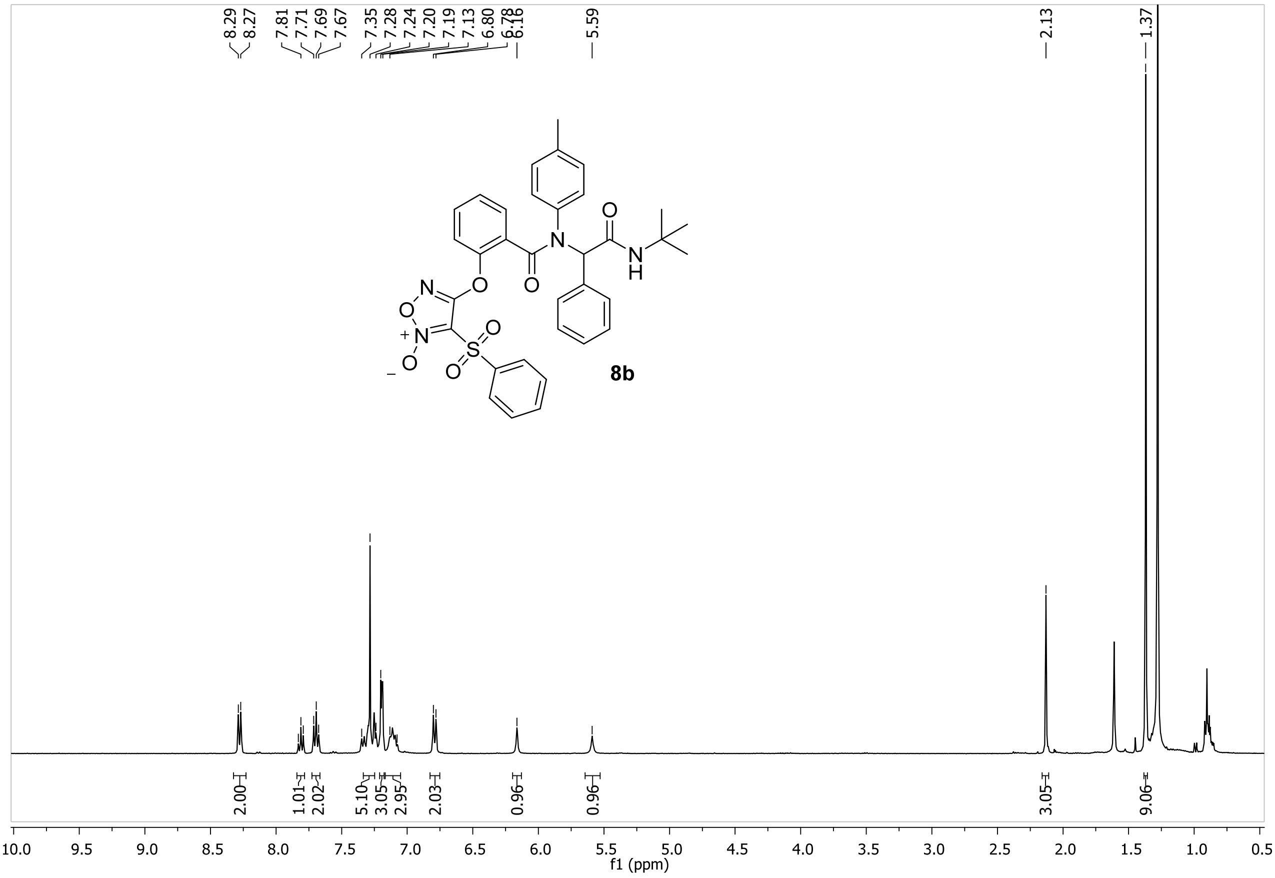
8.29
8.27
7.81
7.71
7.69
7.67
7.35
7.28
7.24
7.20
7.19
7.13
6.80
6.78
6.16
5.59
2.13
1.37
8b
2.00
1.01
2.02
5.10
3.05
2.95
2.03
0.96
0.96
3.05
9.06
f1 (ppm)

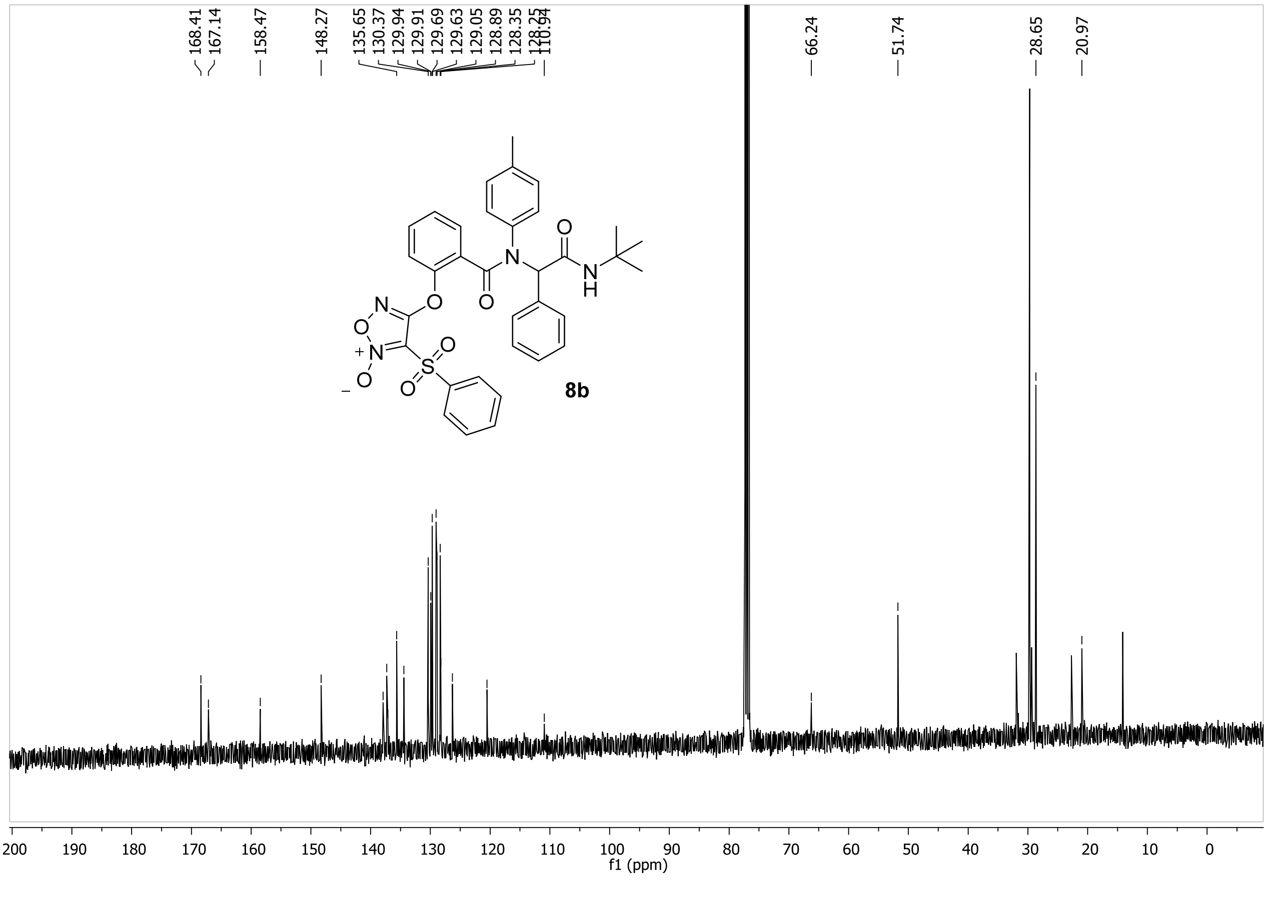
168.41
167.14
158.47
148.27
135.65
130.37
129.94
129.91
129.69
129.63
129.05
128.89
128.35
128.25
110.94
66.24
51.74
28.65
20.97
8b
f1 (ppm)

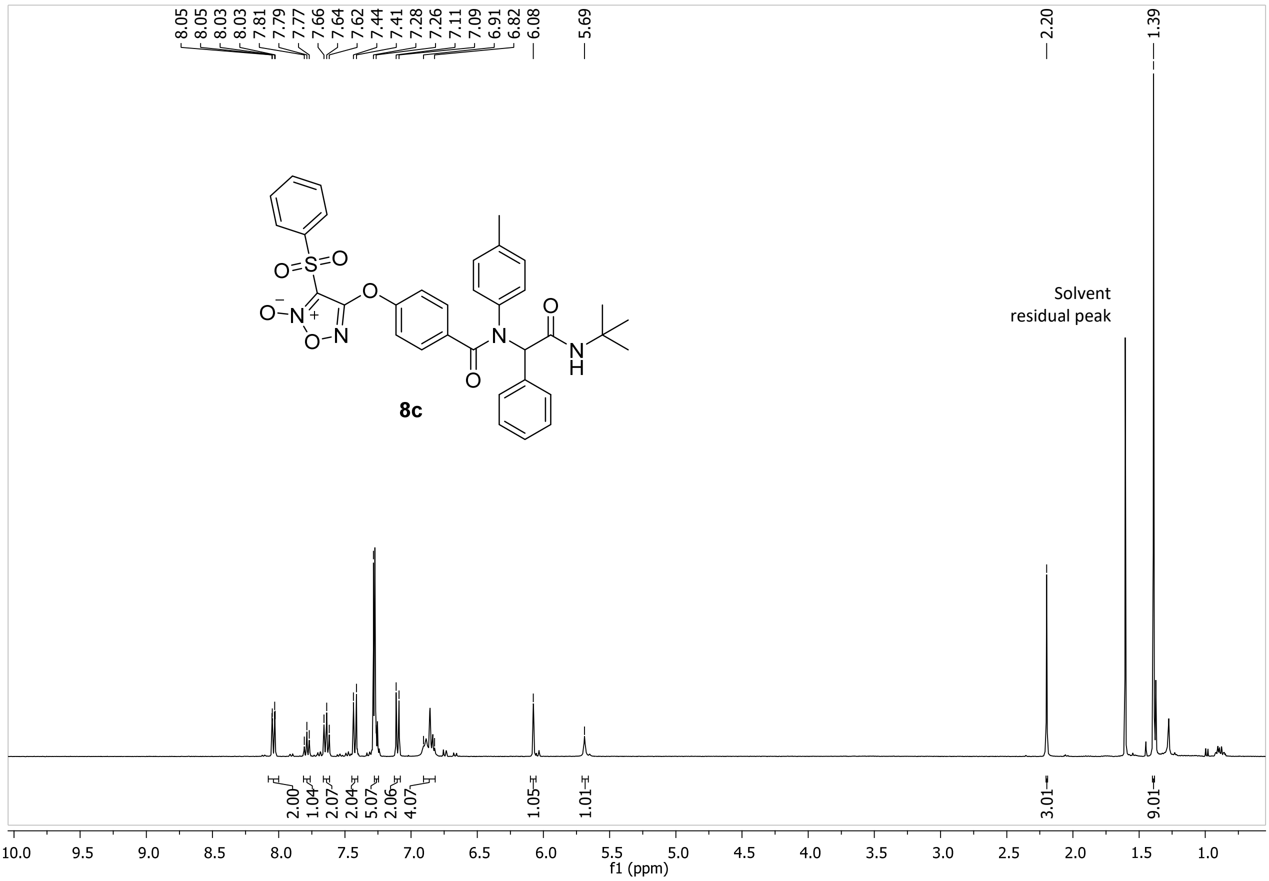
8.05
8.05
8.03
8.03
7.81
7.79
7.77
7.66
7.64
7.62
7.44
7.41
7.28
7.26
7.11
7.09
6.91
6.82
6.08
5.69
2.20
1.39
8c
Solvent
residual peak
2.00
1.04
2.07
2.04
5.07
2.06
4.07
1.05
1.01
3.01
9.01
f1 (ppm)

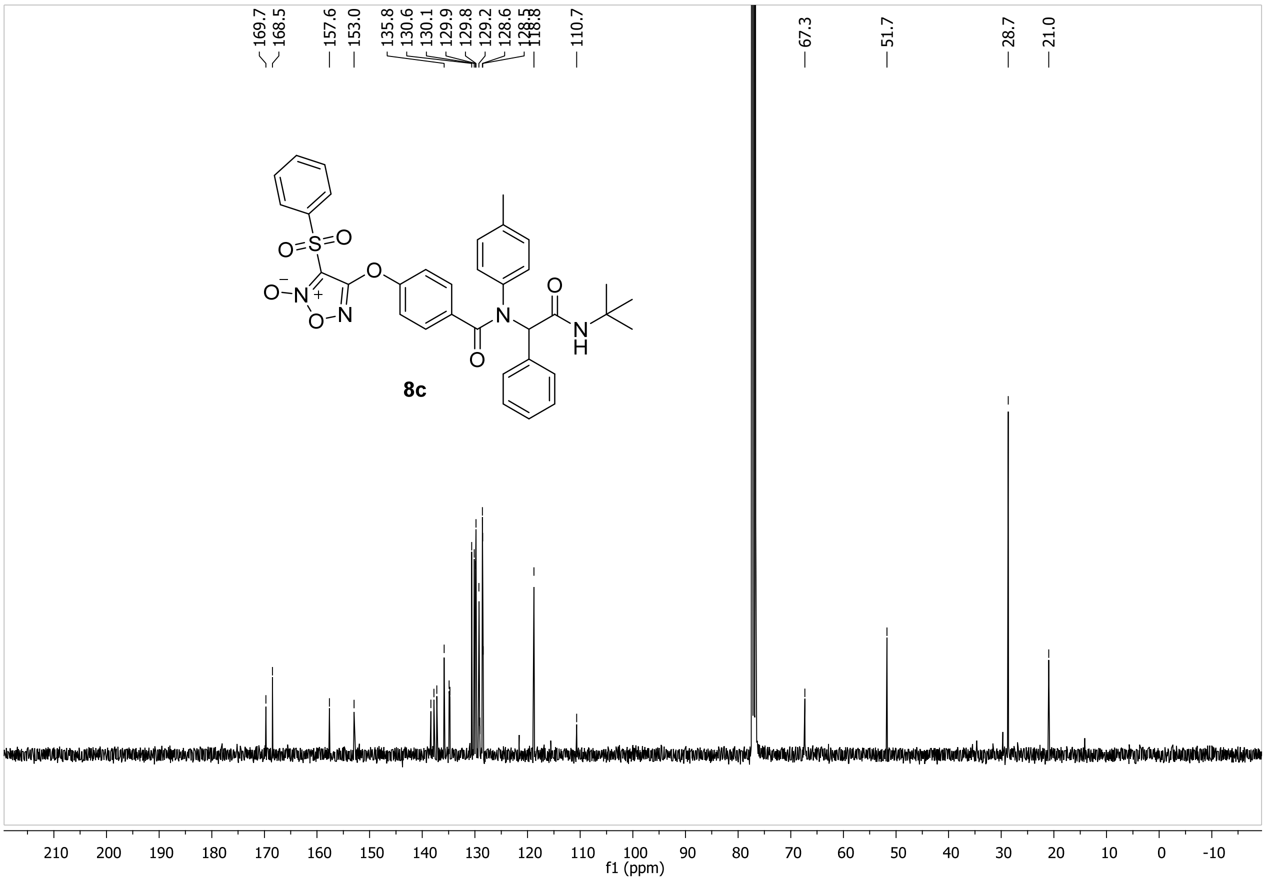
169.7
168.5
157.6
153.0
135.8
130.6
130.1
129.9
129.8
129.2
128.6
128.5
118.8
110.7
67.3
51.7
28.7
21.0
8c
f1 (ppm)

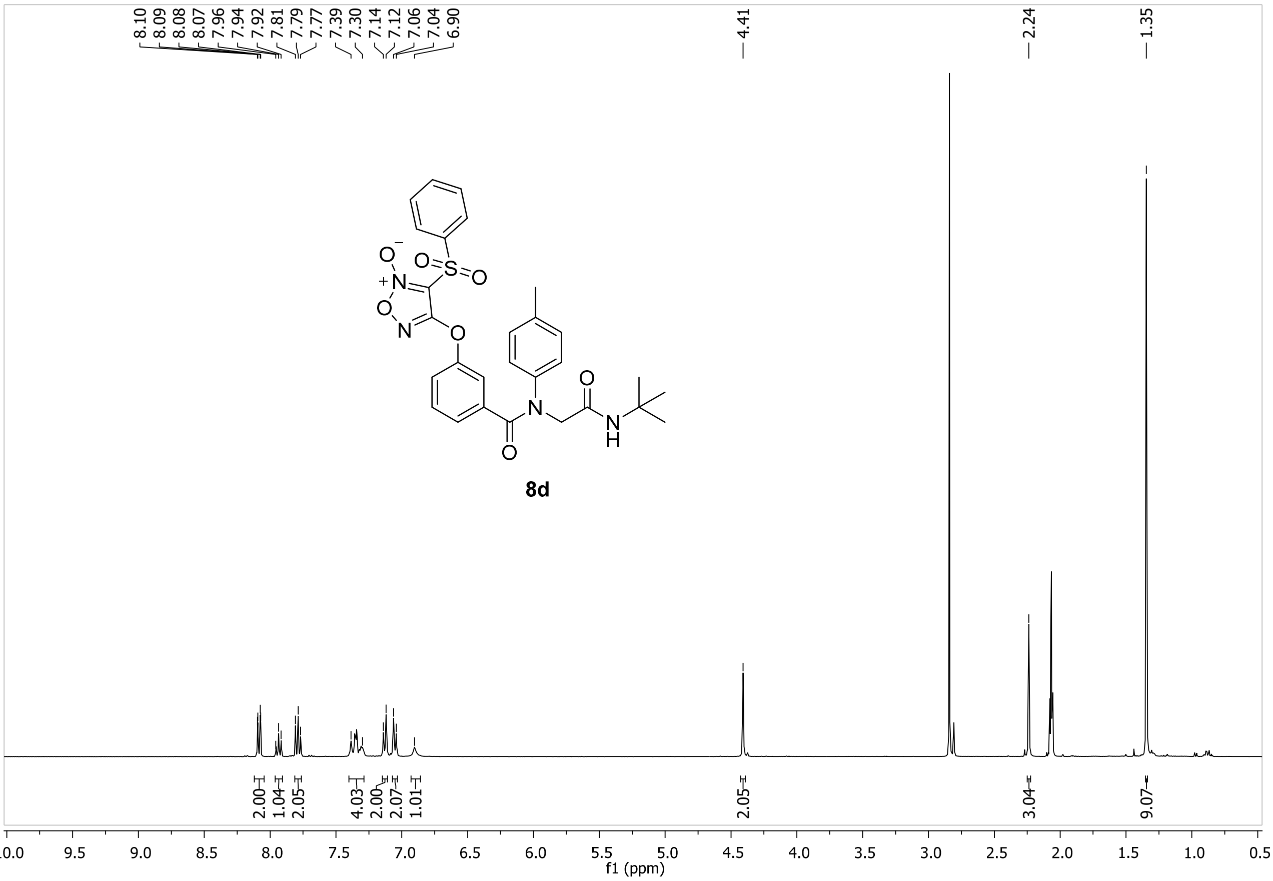
8.10
8.09
8.08
8.07
7.96
7.94
7.92
7.81
7.79
7.77
7.39
7.30
7.14
7.12
7.06
7.04
6.90
4.41
2.24
1.35
8d
2.00
1.04
2.05
4.03
2.00
2.07
1.01
2.05
3.04
9.07
f1 (ppm)

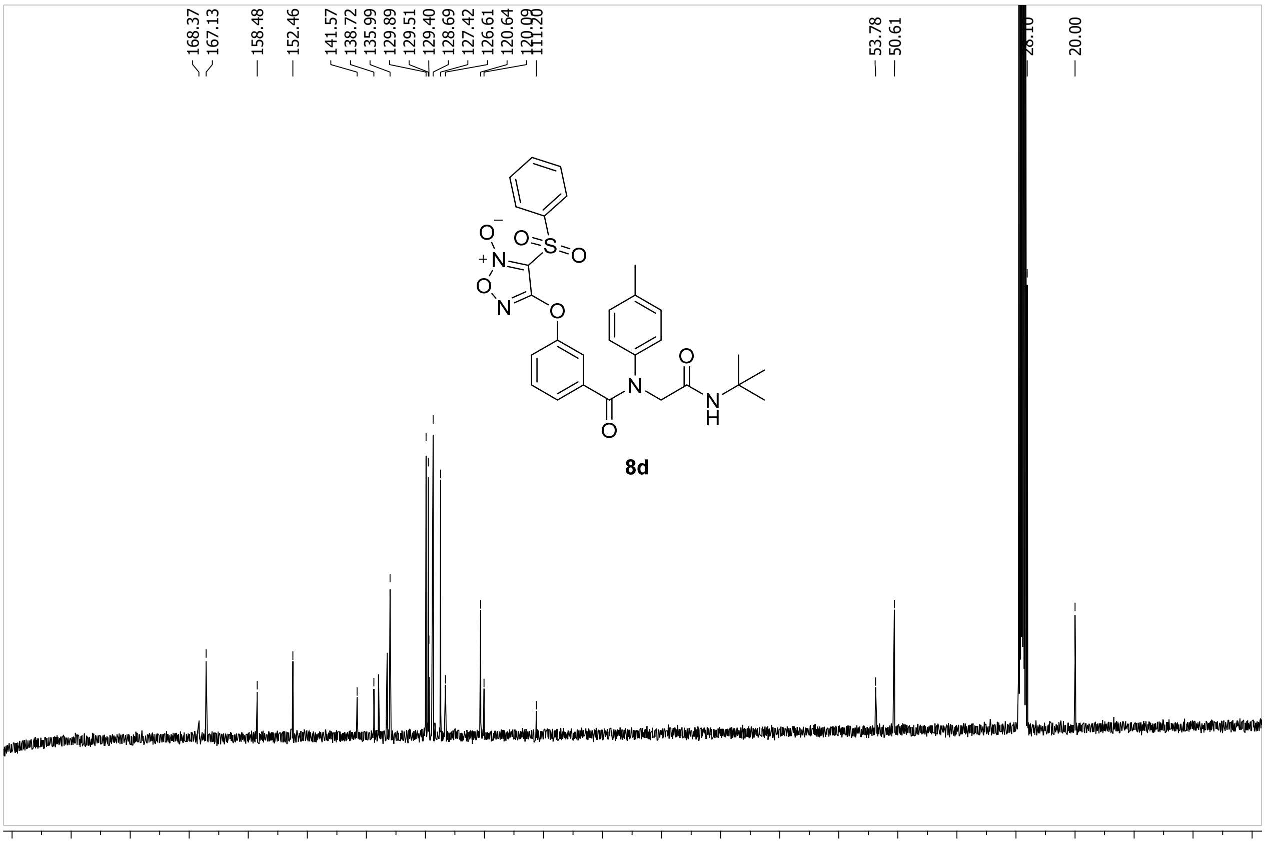
168.37
167.13
158.48
152.46
141.57
138.72
135.99
129.89
129.51
129.40
128.69
127.42
126.61
120.64
120.09
111.20
53.78
50.61
28.10
20.00
8d
f1 (ppm)

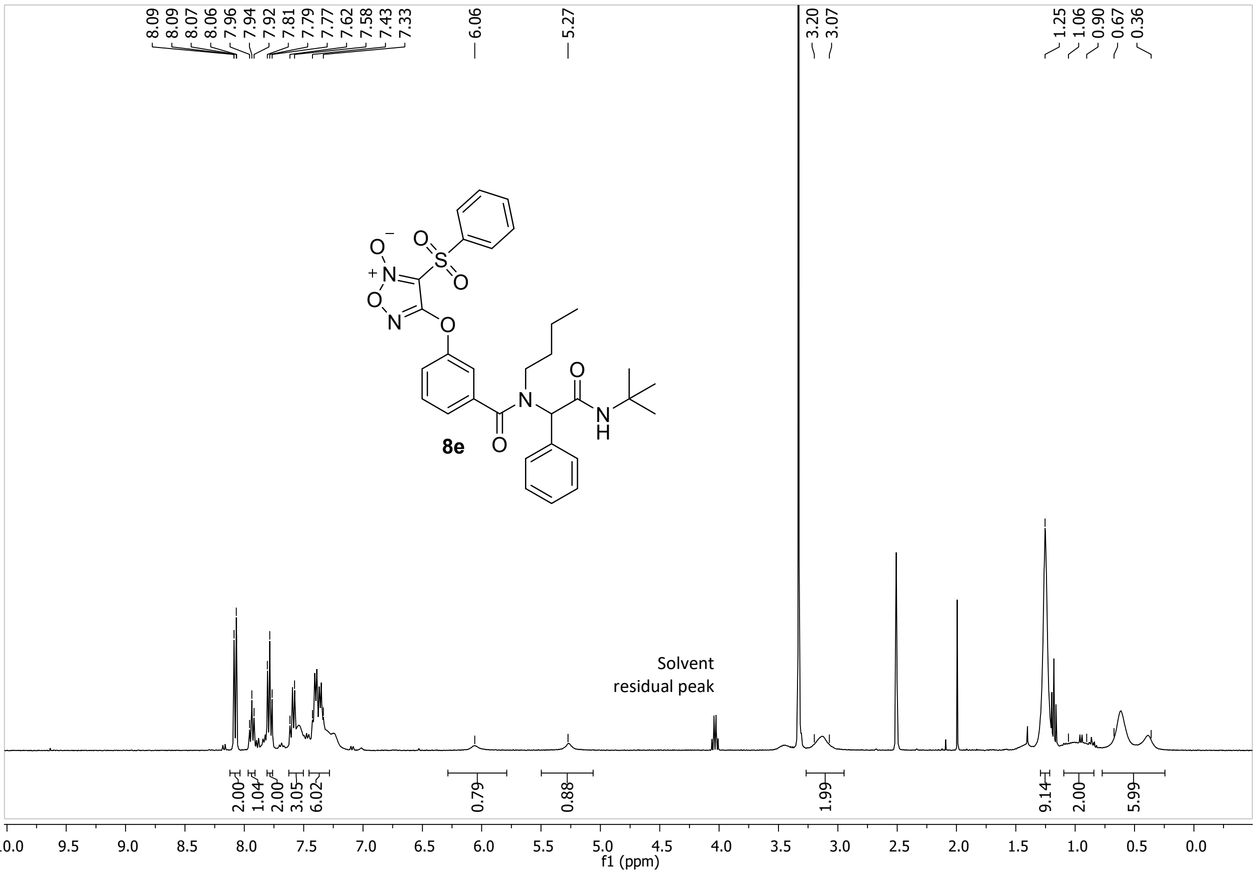
8.09 8.09 8.07 8.06 7.96 7.94 7.92 7.81 7.79 7.77 7.62 7.58 7.43 7.33
6.06
5.27
3.20 3.07
1.25 1.06 0.90 0.67 0.36
8e
Solvent residual peak
2.00 1.04 2.00 3.05 6.02
0.79
0.88
1.99
9.14 2.00 5.99
f1 (ppm)

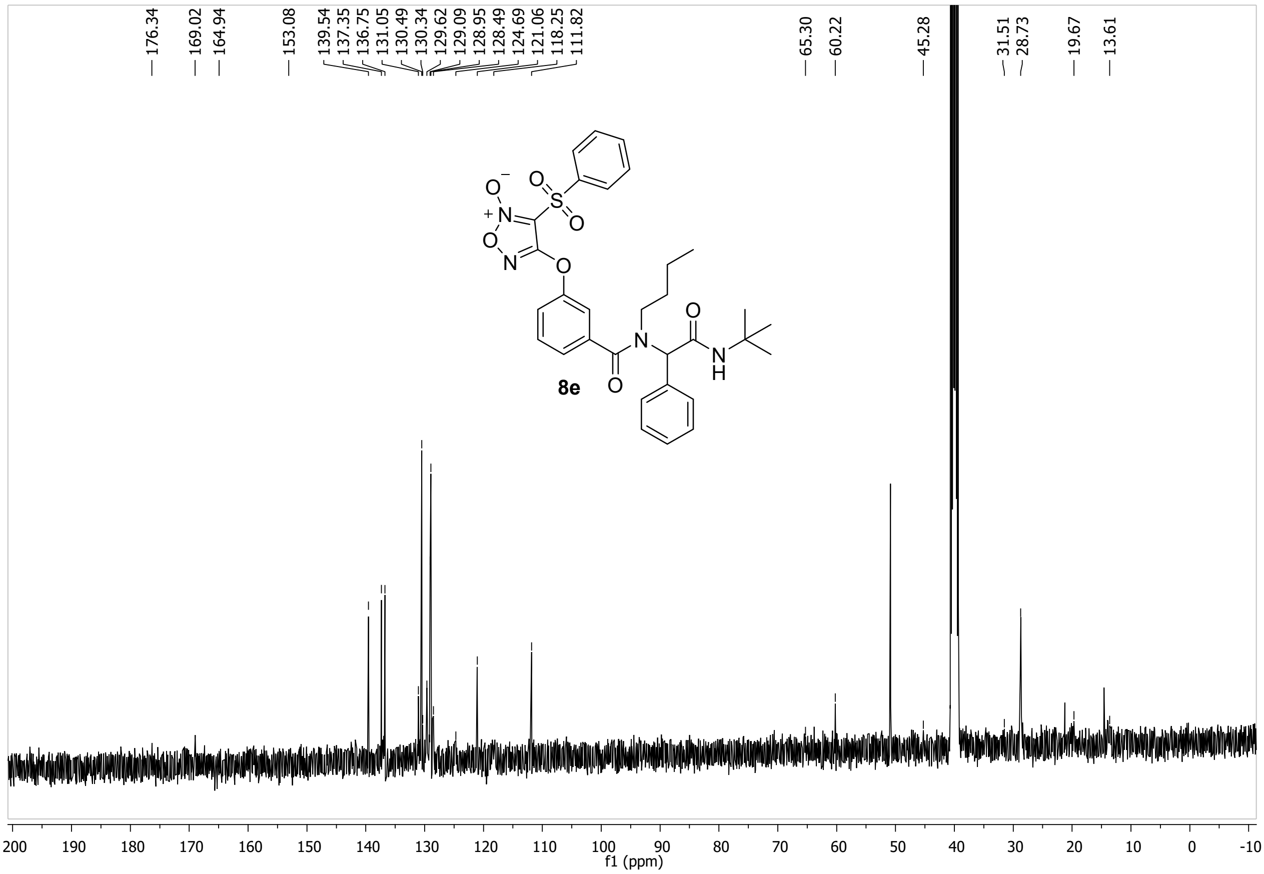
176.34
169.02
164.94
153.08
139.54 137.35 136.75 131.05 130.49 130.34 129.62 129.09 128.95 128.49 124.69 121.06 118.25 111.82
65.30
60.22
45.28
31.51 28.73
19.67
13.61
8e
f1 (ppm)

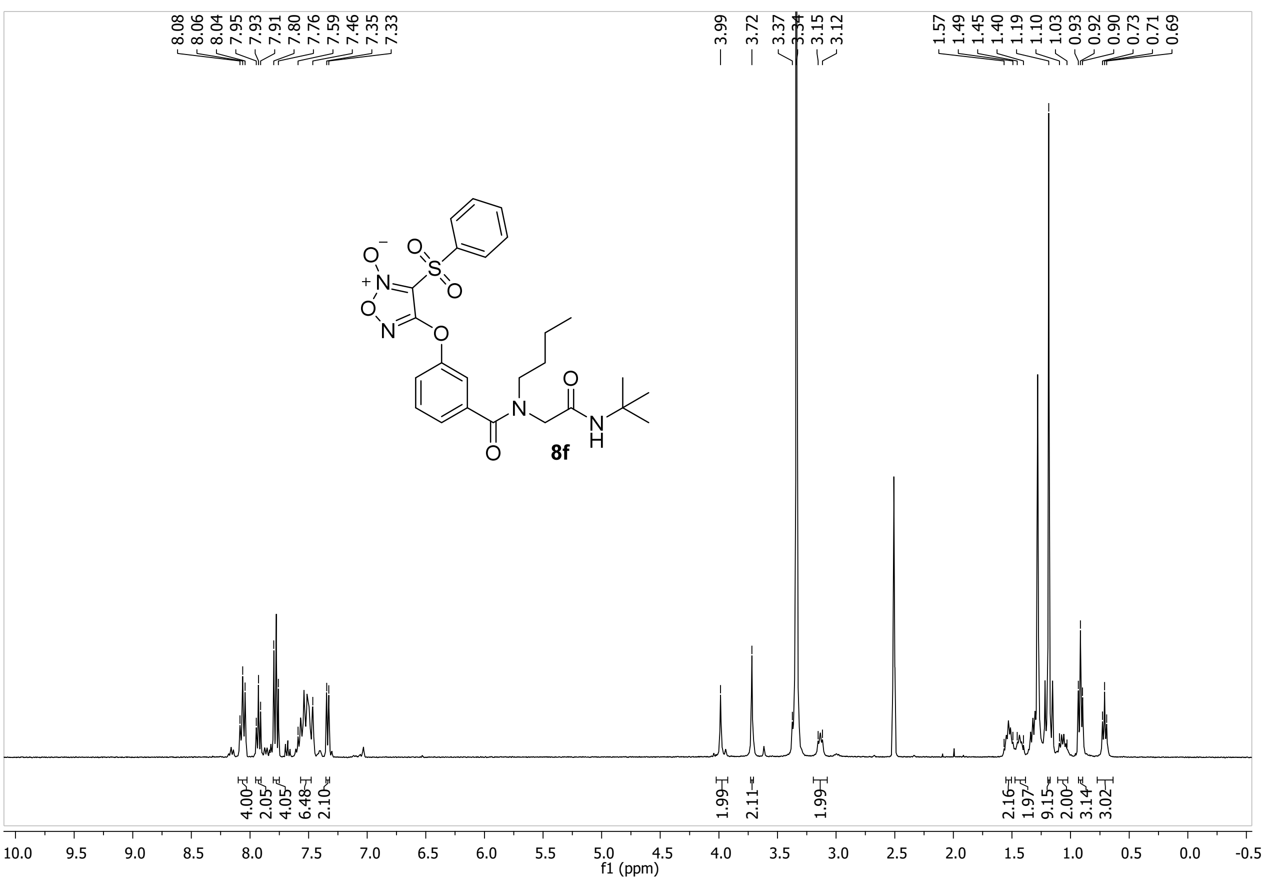
8.08
8.06
8.04
7.95
7.93
7.91
7.80
7.76
7.59
7.46
7.35
7.33
3.99
3.72
3.37
3.34
3.15
3.12
1.57
1.49
1.45
1.40
1.19
1.10
1.03
0.93
0.92
0.90
0.73
0.71
0.69
8f
4.00
2.05
4.05
6.48
2.10
1.99
2.11
1.99
2.16
1.97
9.15
2.00
3.14
3.02
f1 (ppm)

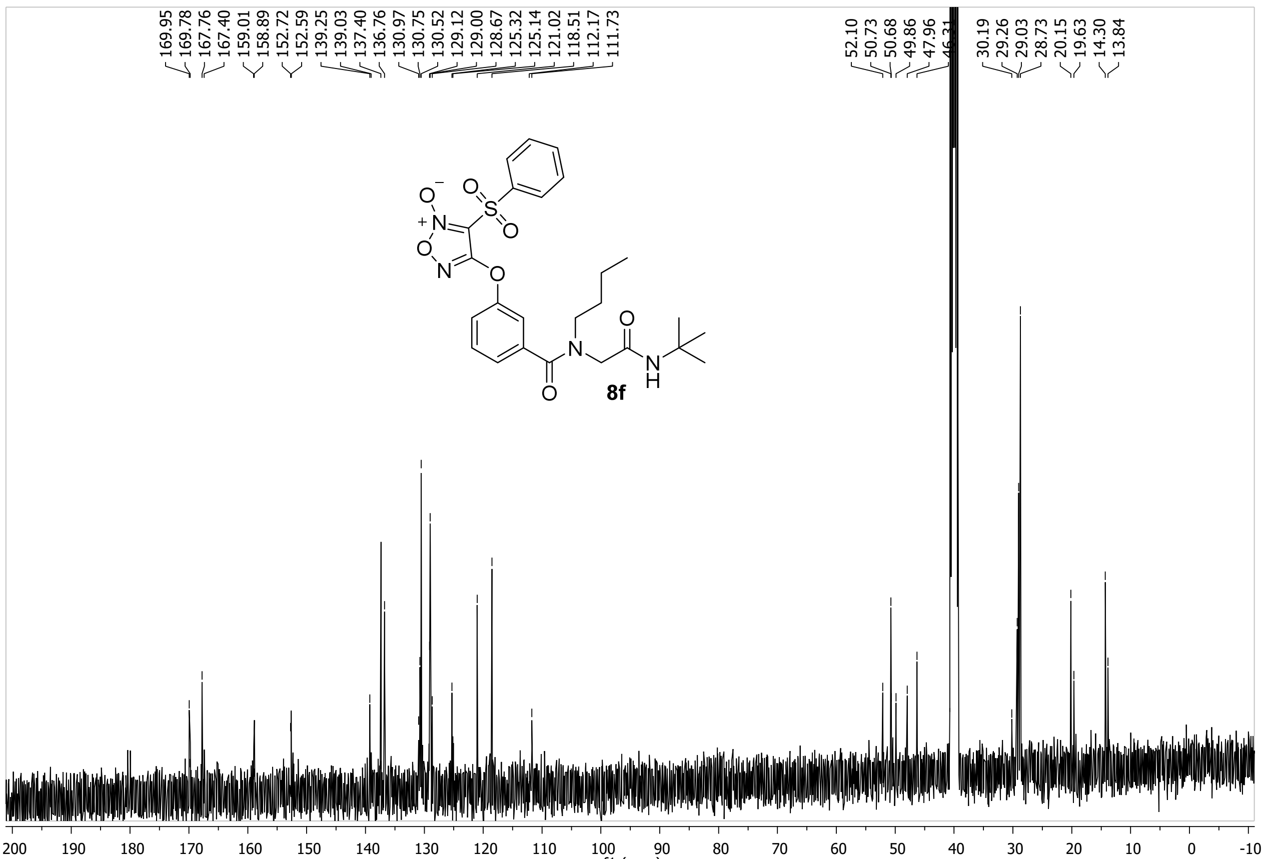
169.95
169.78
167.76
167.40
159.01
158.89
152.72
152.59
139.25
139.03
137.40
136.76
130.97
130.75
130.52
129.12
129.00
128.67
125.32
125.14
121.02
118.51
112.17
111.73
52.10
50.73
50.68
49.86
47.96
30.19
29.26
29.03
28.73
20.15
19.63
14.30
13.84
8f
f1 (ppm)

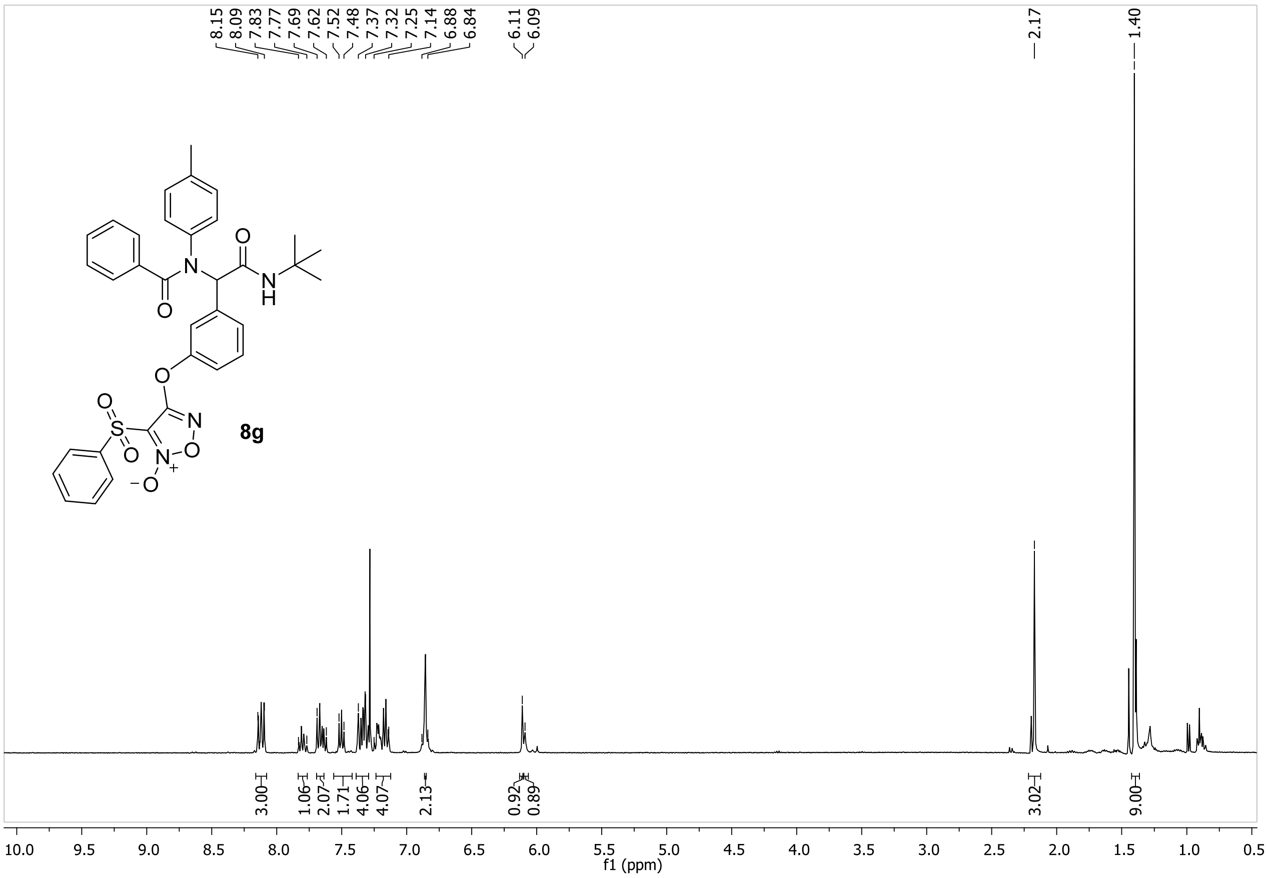

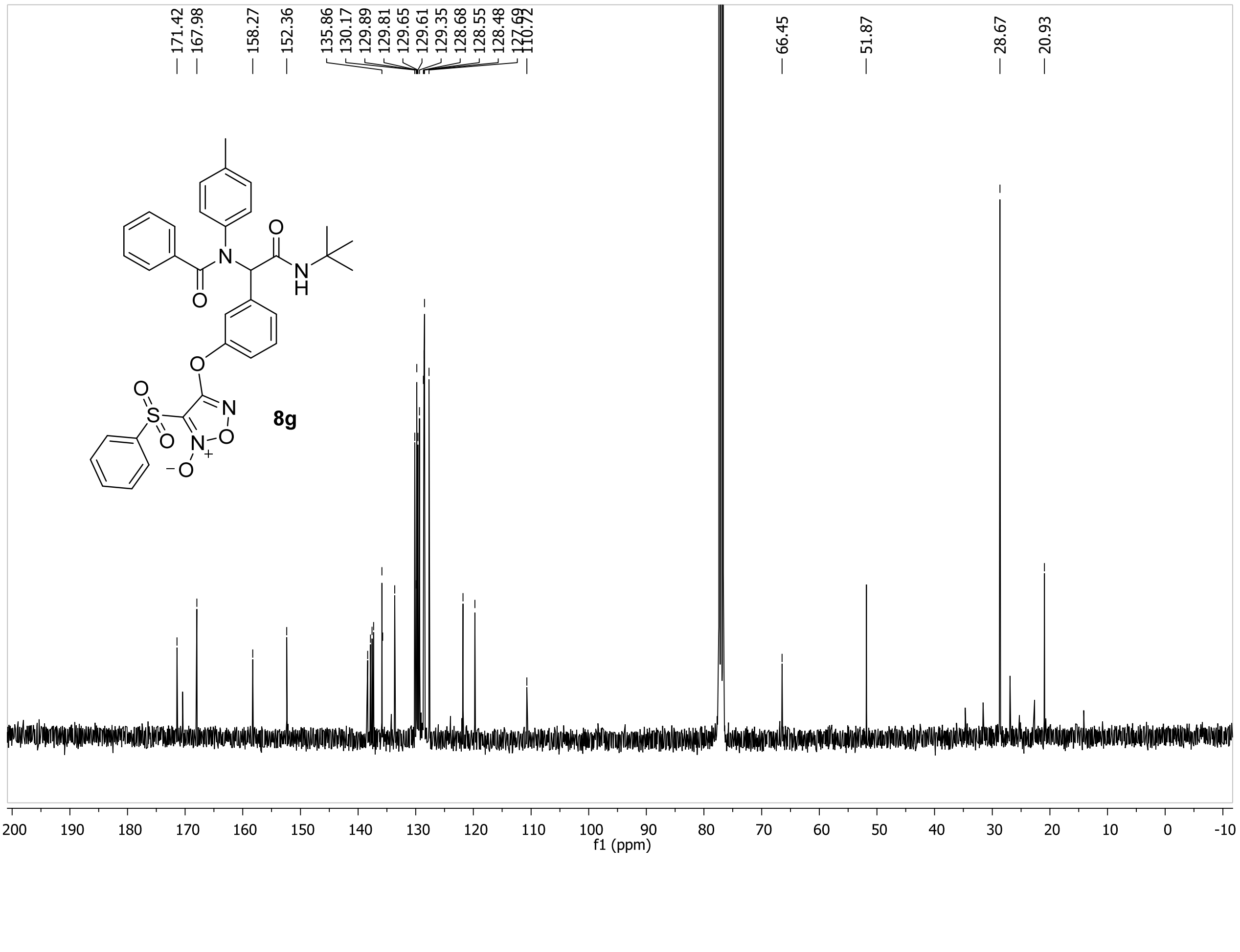

GBB reaction derivatives

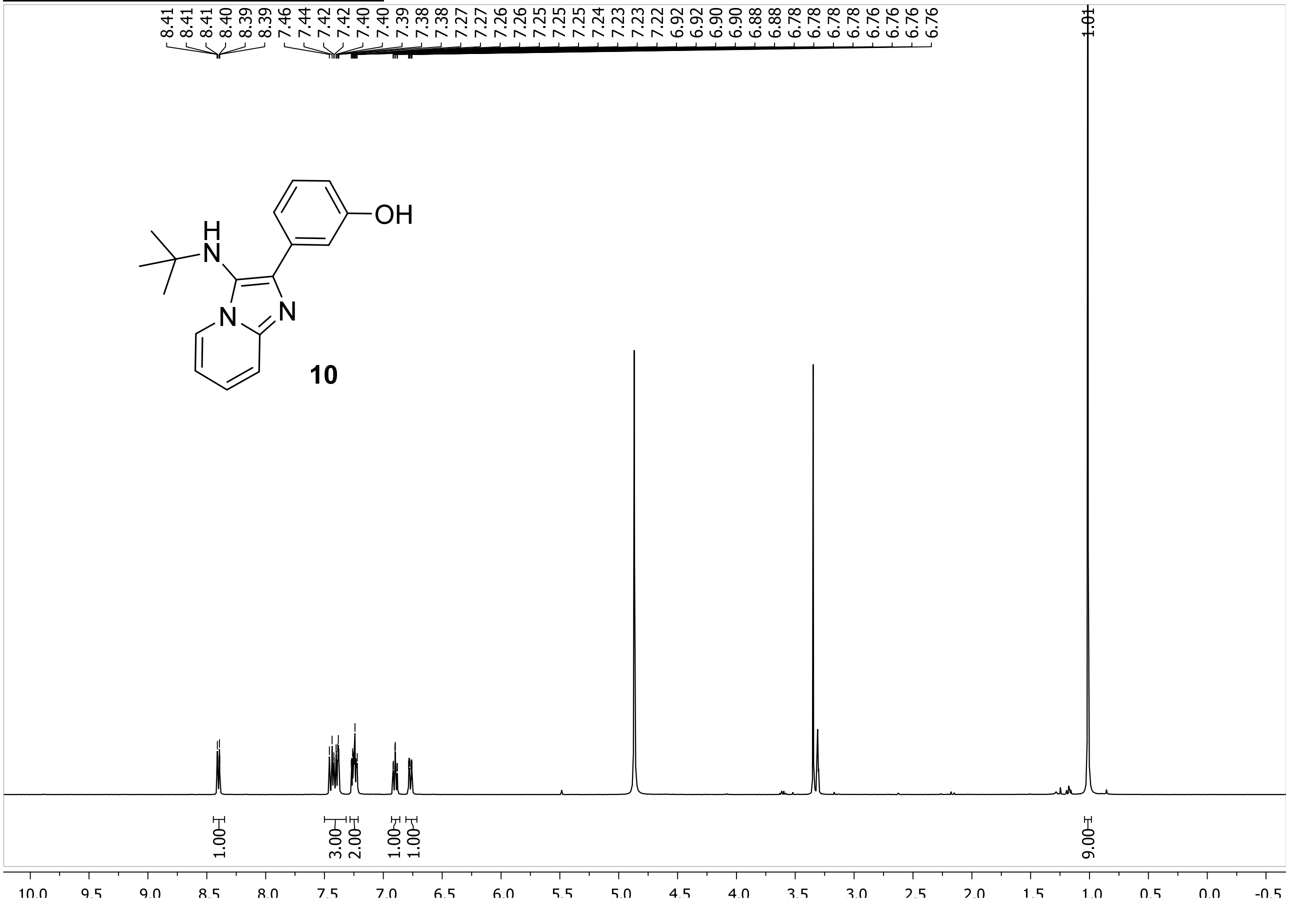

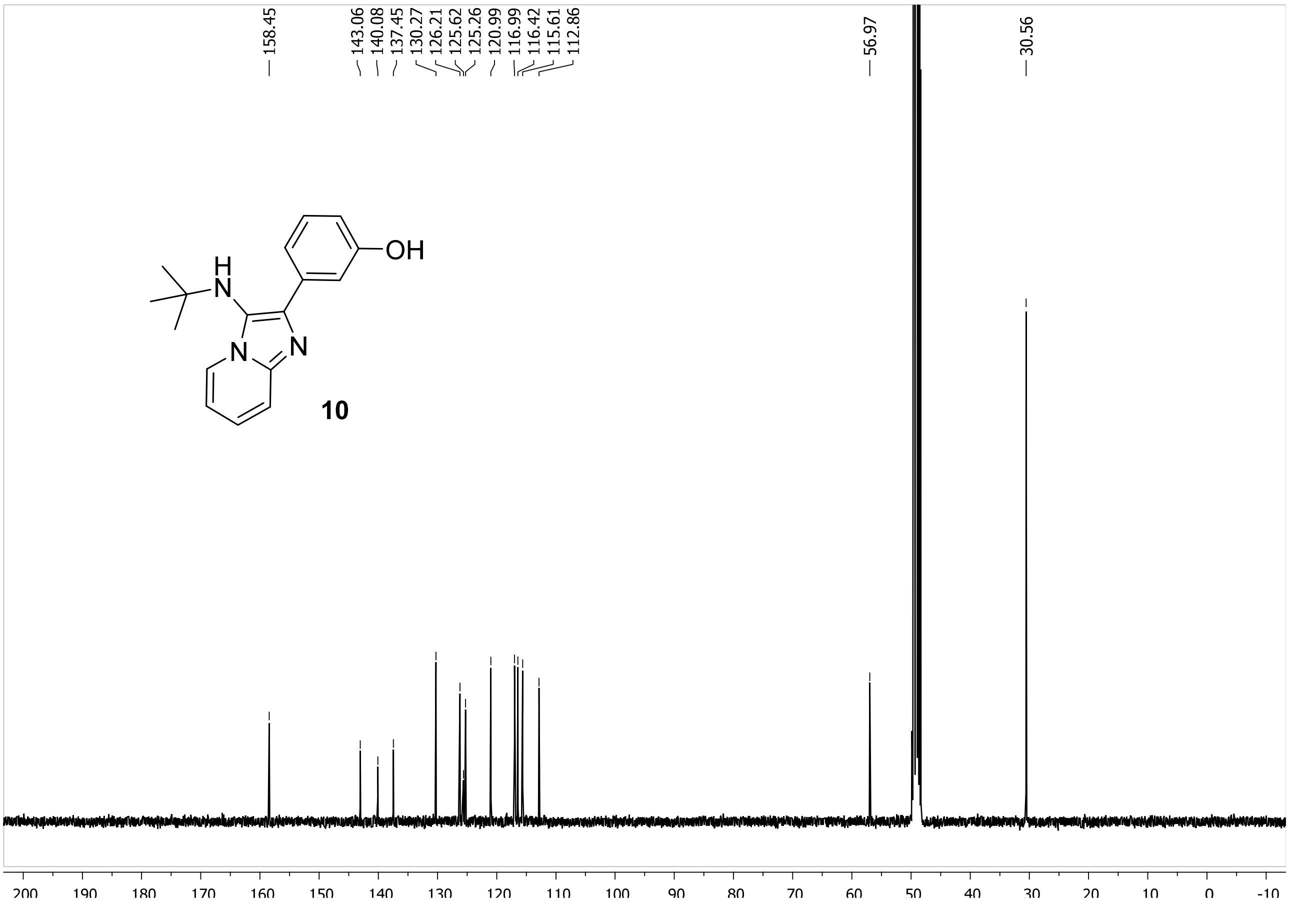

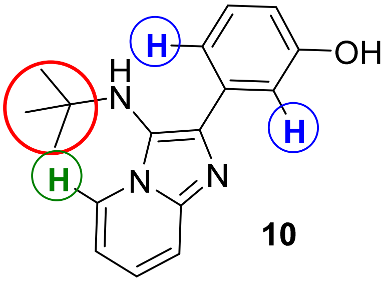

10

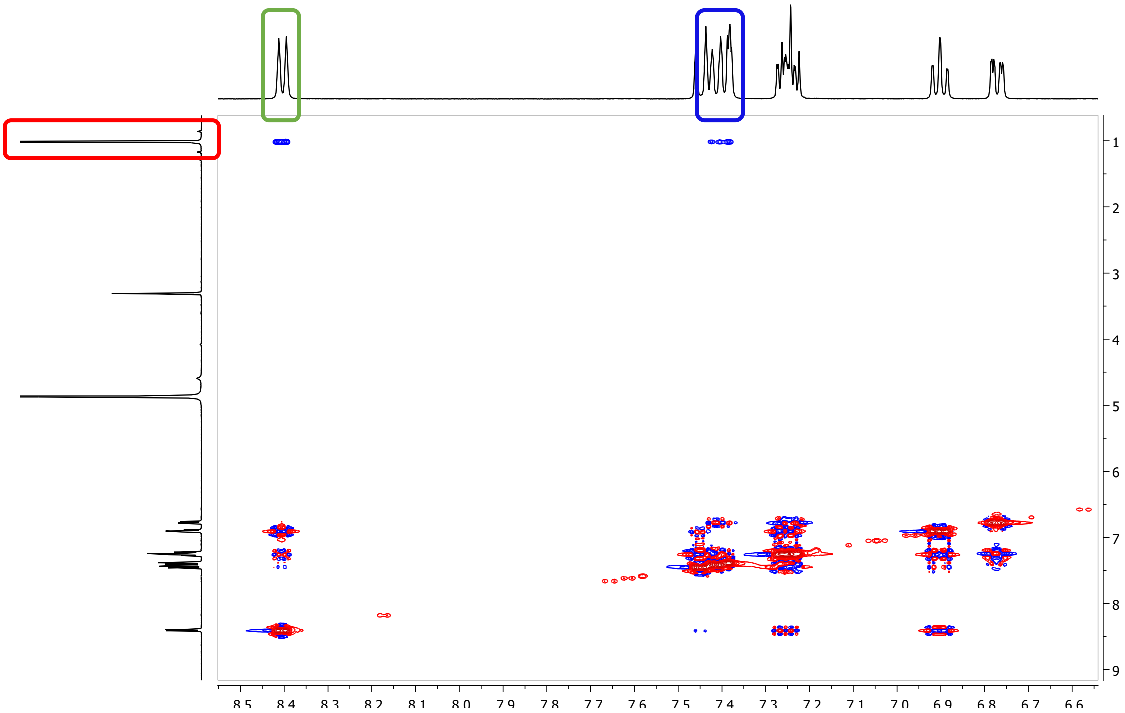

2D-NOESY-NMR spectrum in methanol-d4 of compound **10**. Cross coupling signals of the *tert*-butylisocyanide group protons (red) with the aromatic protons (blue) and the *ortho* proton (green) in the pyridine ring are highlighted.

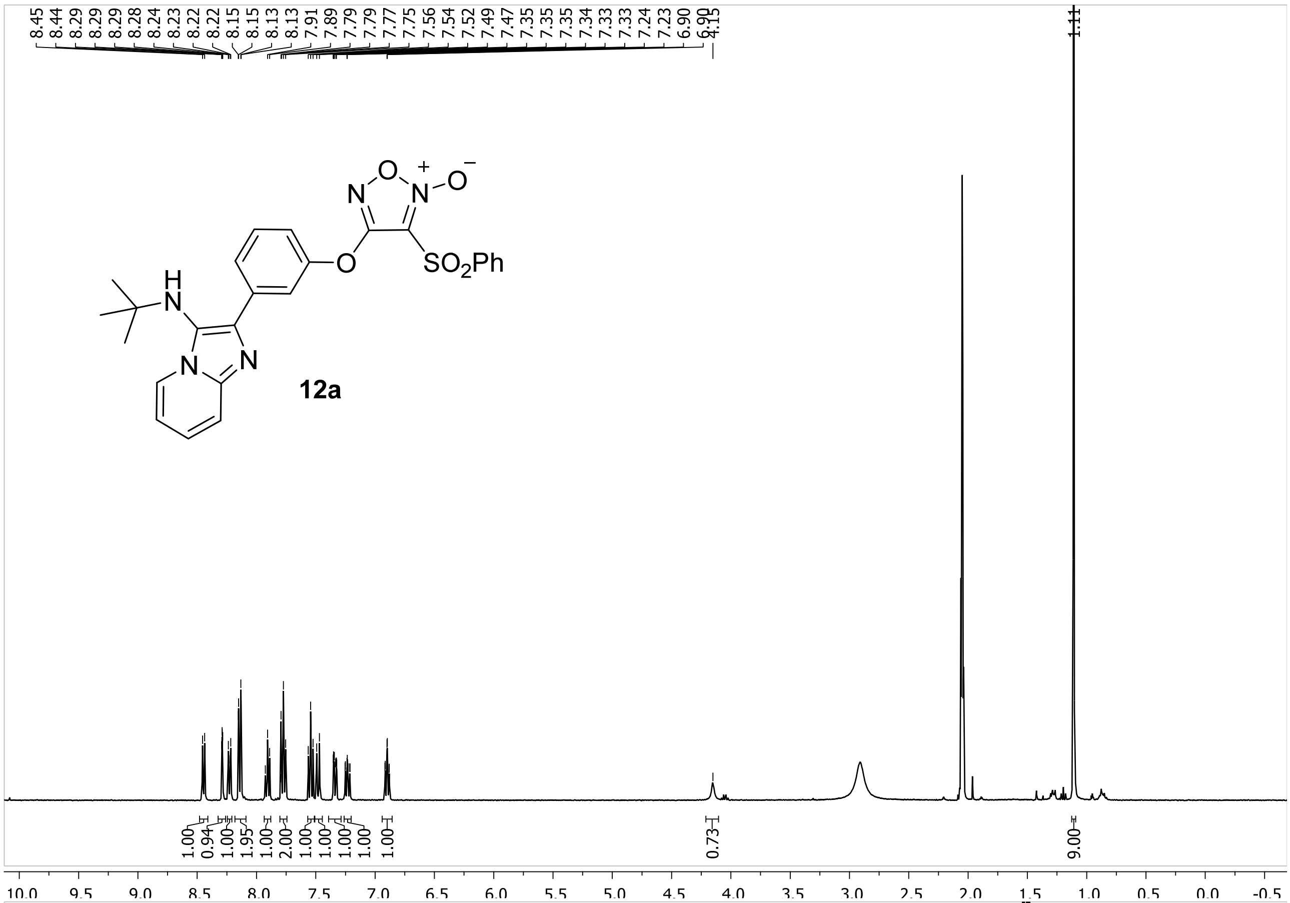

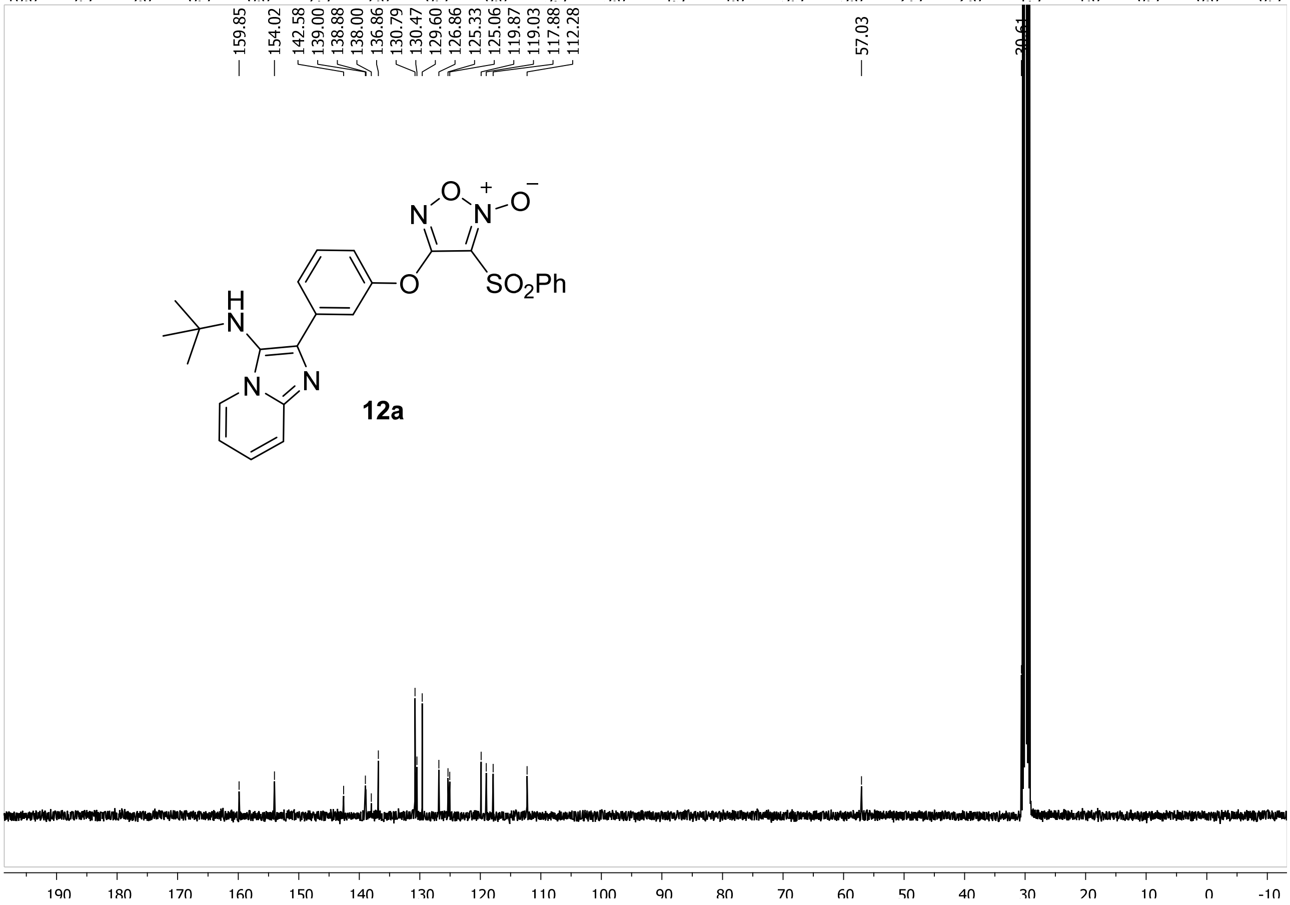

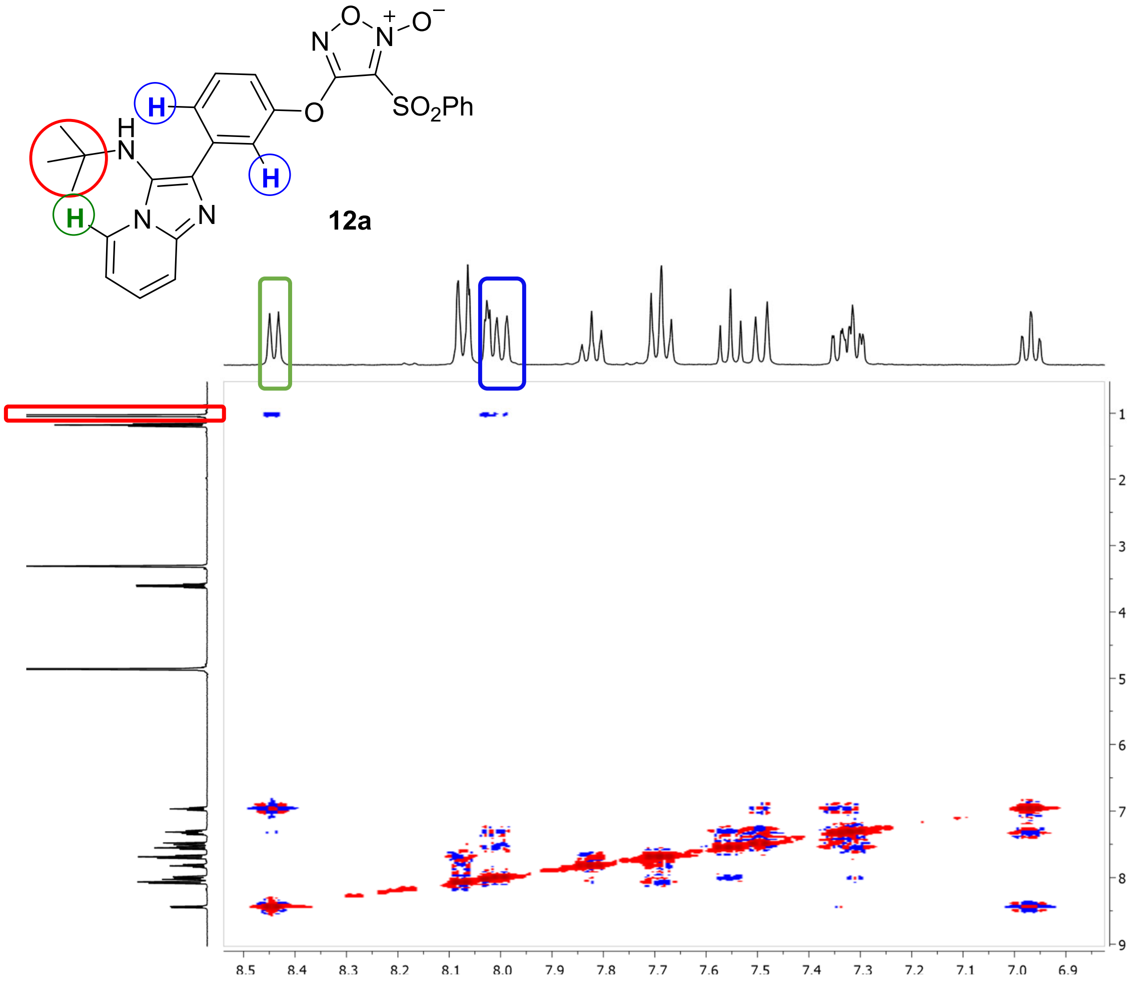

2D-NOESY-NMR spectrum in methanol-d4 of compound **12a**. Cross coupling signals of the *tert*-butylisocyanide group protons (red) with the aromatic protons (blue) and the *ortho* proton (green) in the pyridine ring are highlighted.

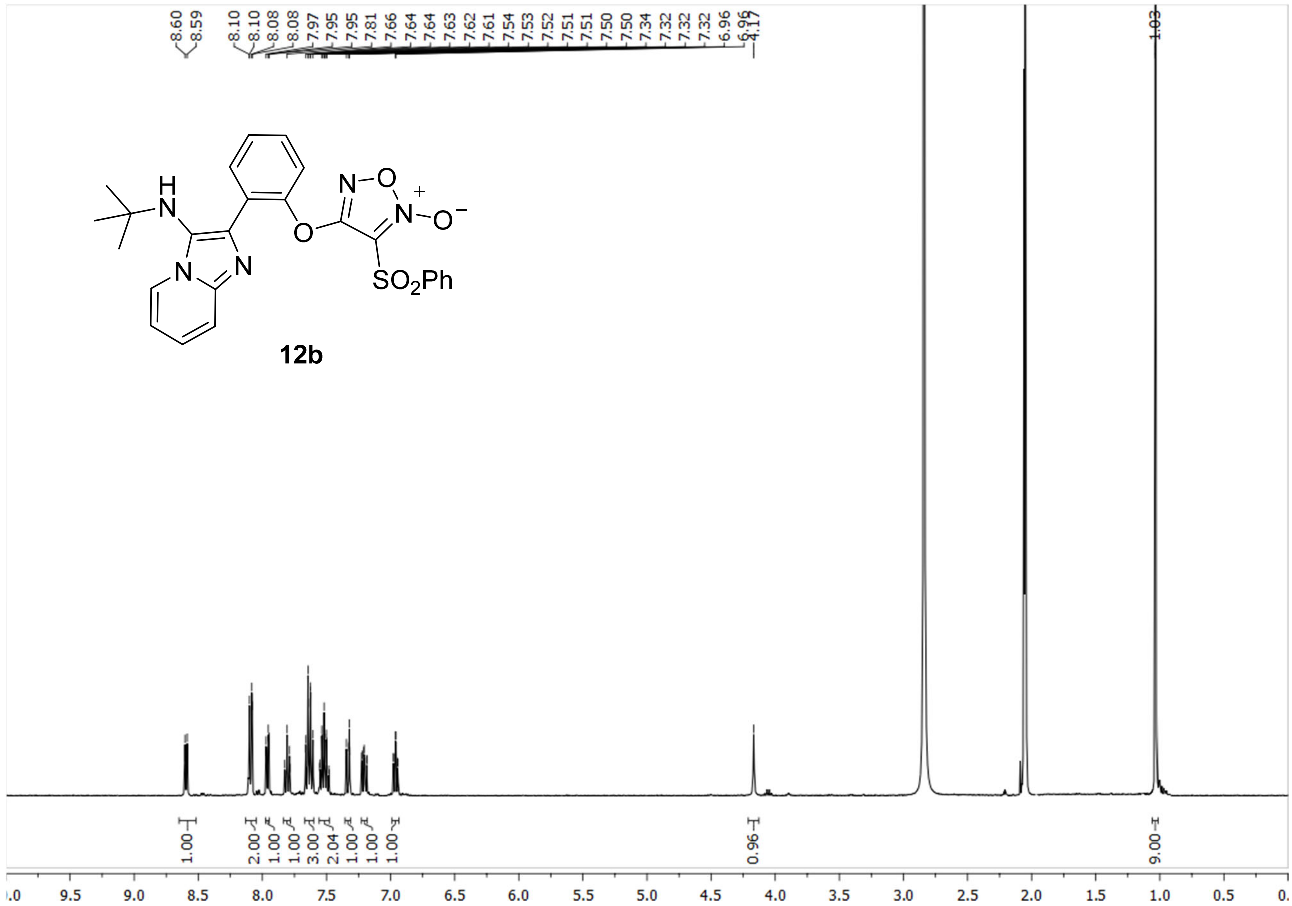

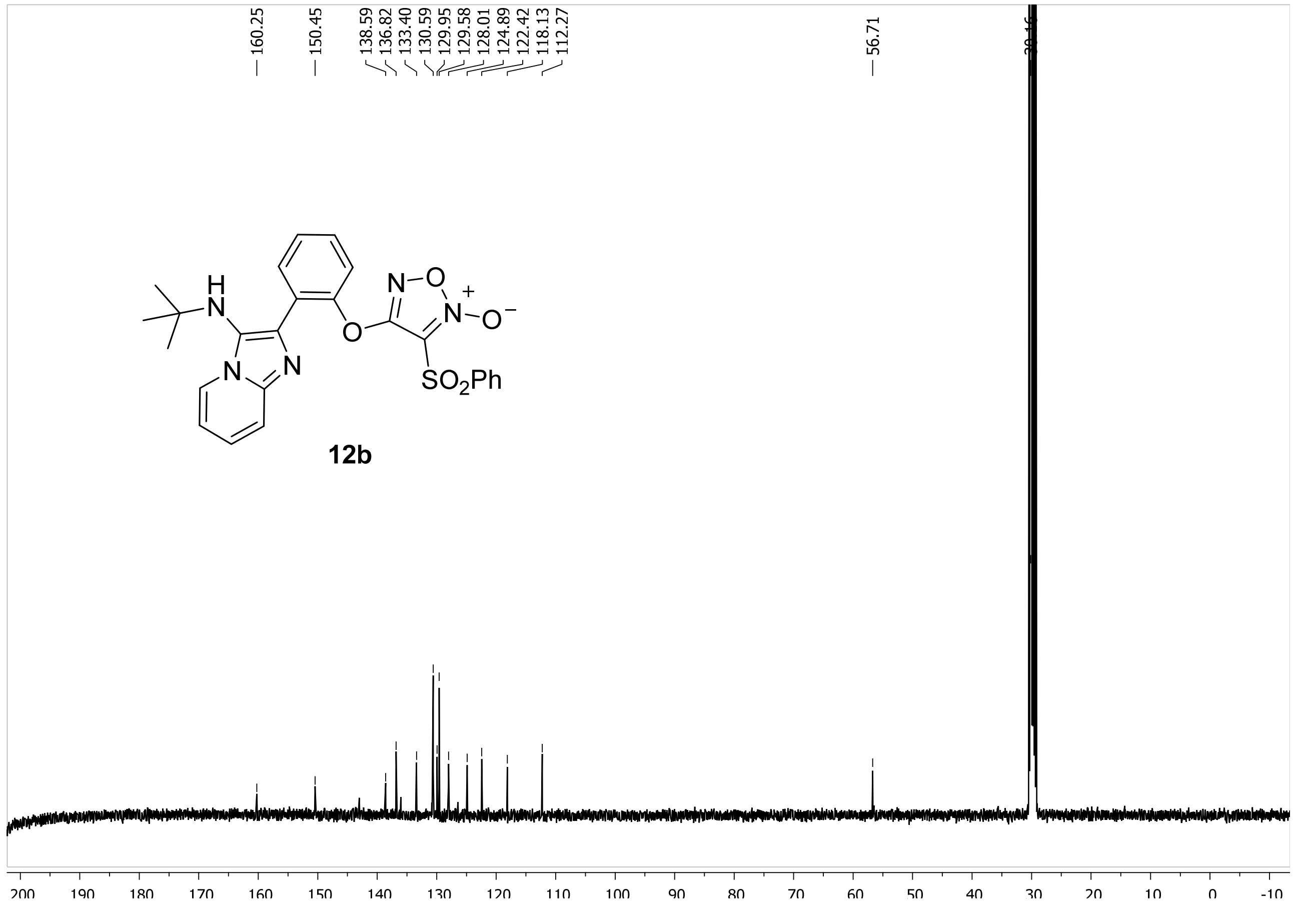

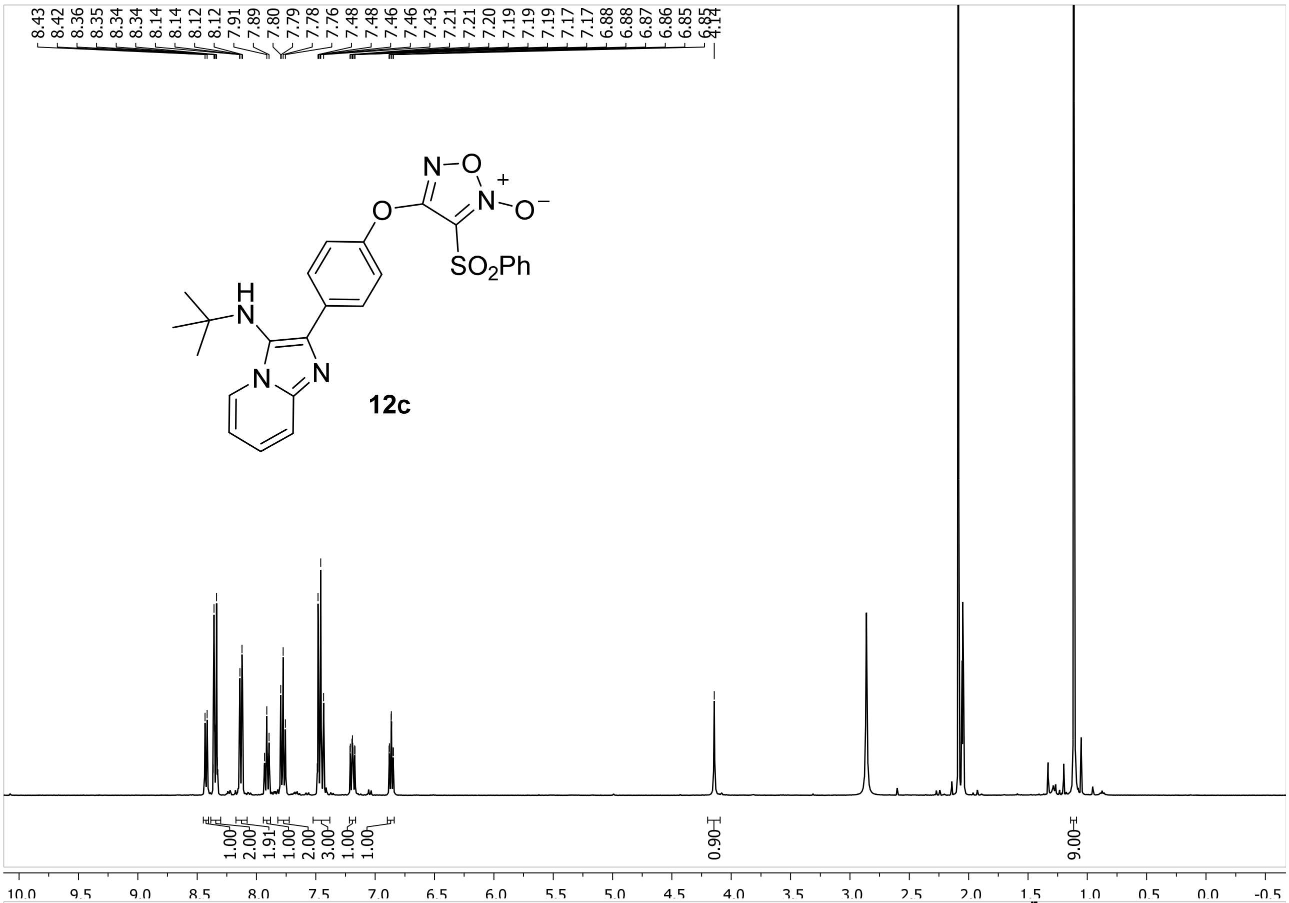
8.43
8.42
8.36
8.35
8.34
8.34
8.14
8.14
8.12
8.12
7.91
7.89
7.80
7.79
7.78
7.76
7.48
7.48
7.46
7.46
7.43
7.21
7.21
7.20
7.19
7.19
7.19
7.17
7.17
6.88
6.88
6.87
6.86
6.85
6.85
4.14
12c
1.00
2.00
1.91
1.00
2.00
3.00
1.00
1.00
0.90
9.00
10.0 9.5 9.0 8.5 8.0 7.5 7.0 6.5 6.0 5.5 5.0 4.5 4.0 3.5 3.0 2.5 2.0 1.5 1.0 0.5 0.0 -0.5

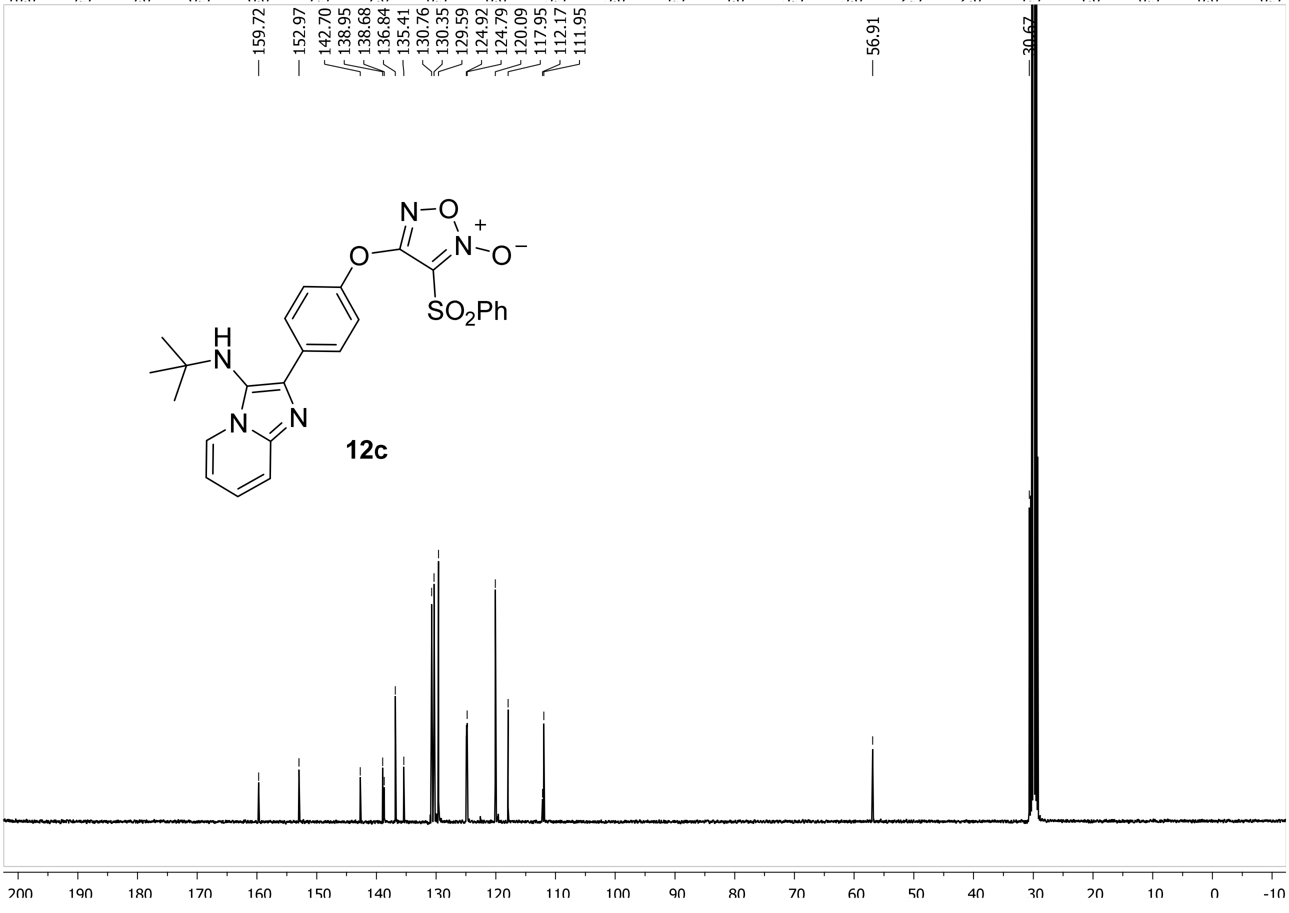
159.72
152.97
142.70
138.95
138.68
136.84
135.41
130.76
130.35
129.59
124.92
124.79
120.09
117.95
112.17
111.95
56.91
30.67
12c
200 190 180 170 160 150 140 130 120 110 100 90 80 70 60 50 40 30 20 10 0 -10

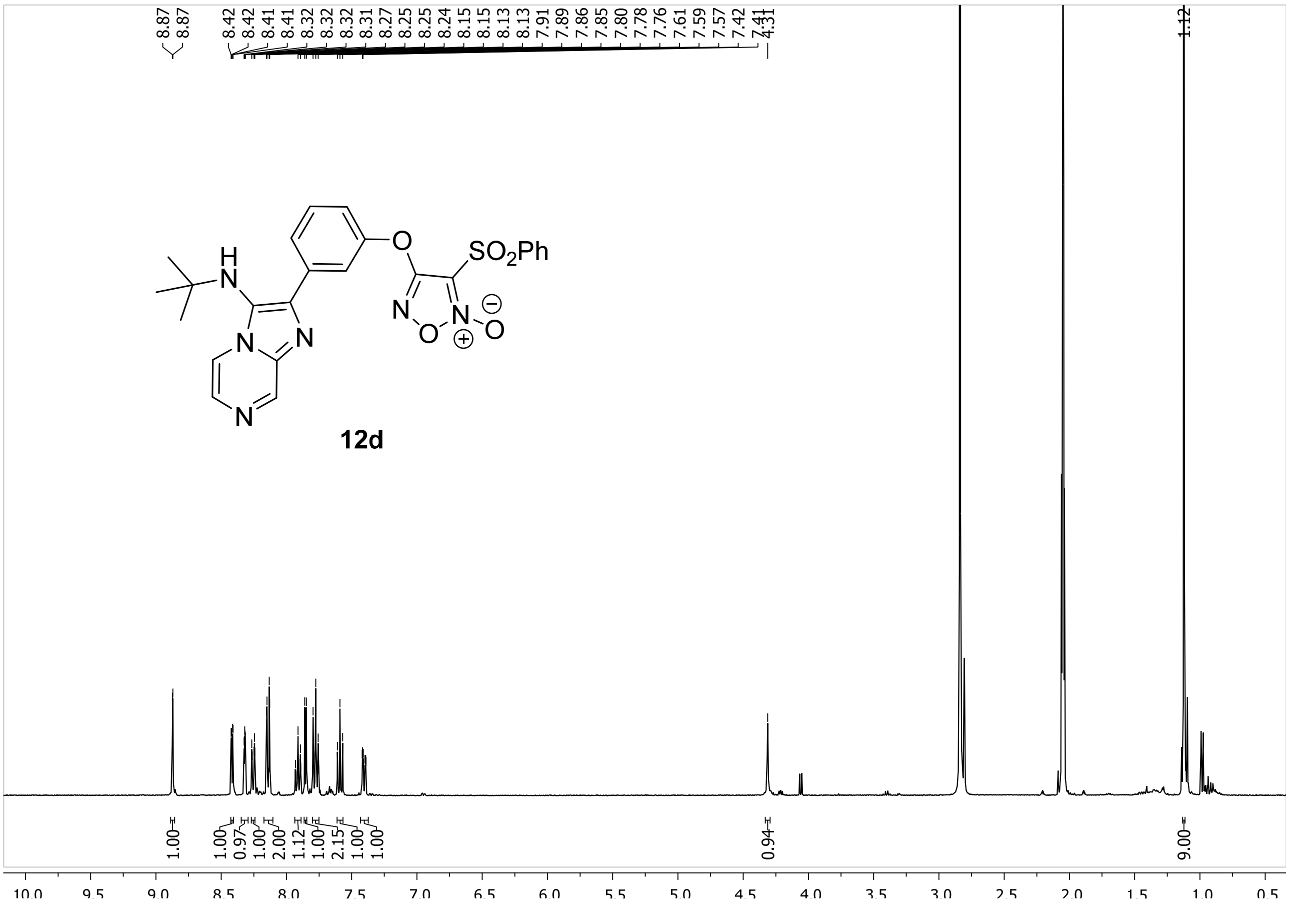
8.87
8.87
8.42
8.42
8.41
8.41
8.32
8.32
8.32
8.31
8.27
8.25
8.25
8.24
8.15
8.15
8.13
8.13
7.91
7.89
7.86
7.85
7.80
7.78
7.76
7.61
7.59
7.57
7.42
7.41
4.31
1.12
H
N
N
N
N
O
SO_2Ph
N
N
O
O
12d
1.00
1.00
0.97
1.00
2.00
1.12
1.00
2.15
1.00
1.00
0.94
9.00
10.0 9.5 9.0 8.5 8.0 7.5 7.0 6.5 6.0 5.5 5.0 4.5 4.0 3.5 3.0 2.5 2.0 1.5 1.0 0.5

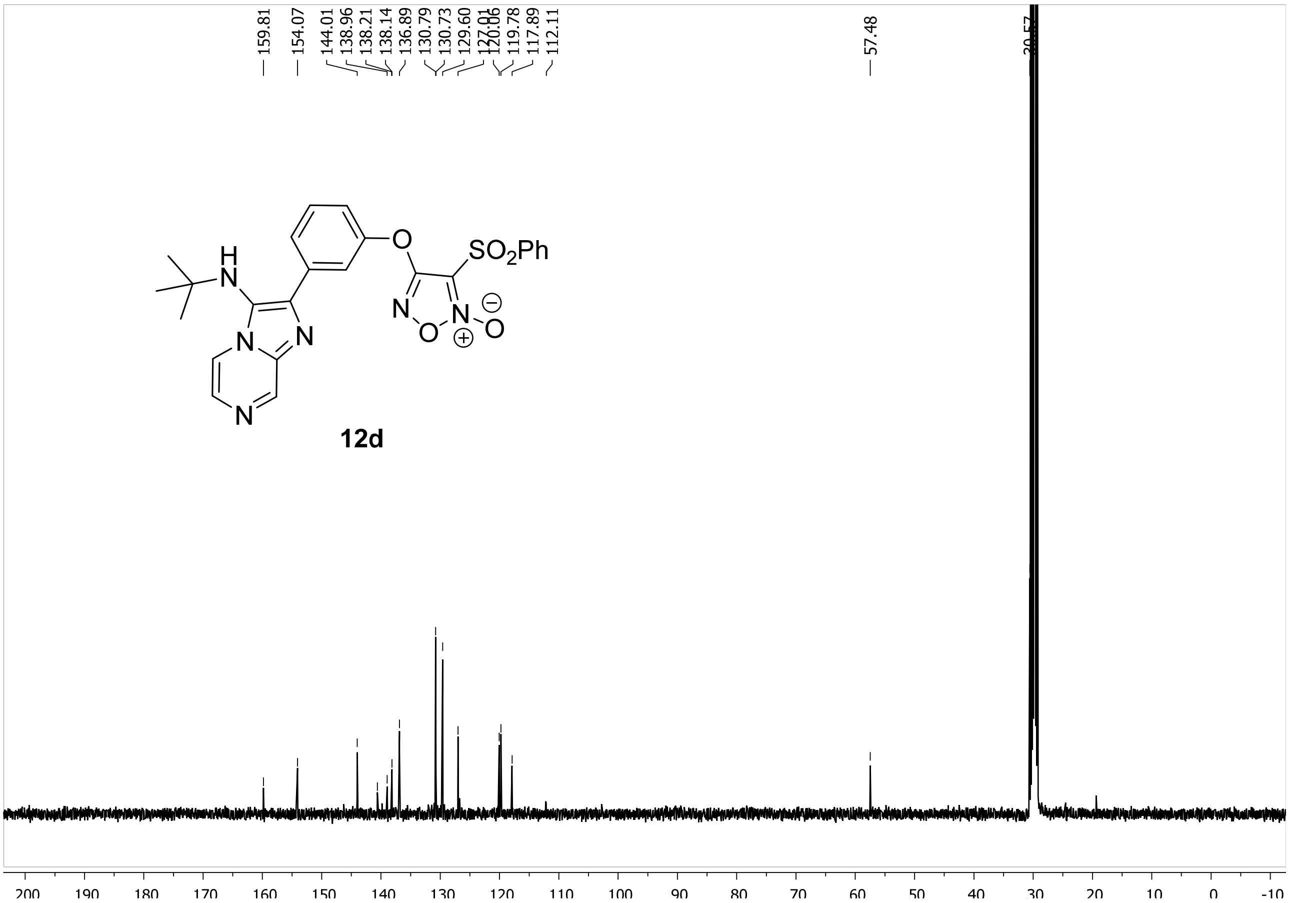
159.81
154.07
144.01
138.96
138.21
138.14
136.89
130.79
130.73
129.60
127.01
120.06
119.78
117.89
112.11
57.48
H
N
N
N
N
O
SO_2Ph
N
N
O
O
12d
200 190 180 170 160 150 140 130 120 110 100 90 80 70 60 50 40 30 20 10 0 -10

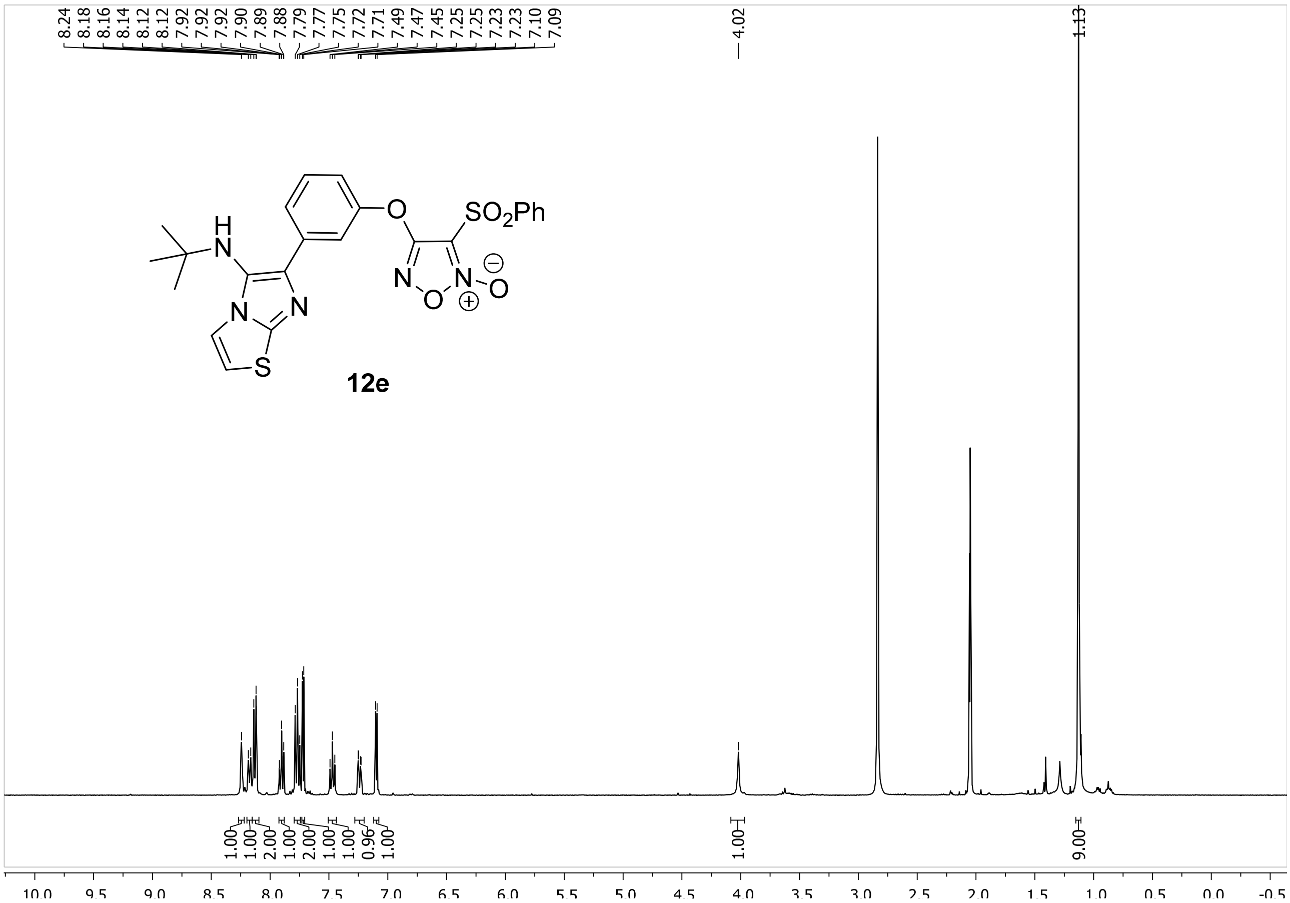
8.24
8.18
8.16
8.14
8.12
8.12
7.92
7.92
7.92
7.90
7.89
7.88
7.79
7.77
7.75
7.72
7.71
7.49
7.47
7.45
7.25
7.25
7.23
7.23
7.10
7.09
4.02
1.13
H
N
O
SO2Ph
N
N
O
O
N
N
S
12e
1.00
1.00
2.00
1.00
2.00
1.00
1.00
0.96
1.00
1.00
9.00
10.0 9.5 9.0 8.5 8.0 7.5 7.0 6.5 6.0 5.5 5.0 4.5 4.0 3.5 3.0 2.5 2.0 1.5 1.0 0.5 0.0 -0.5

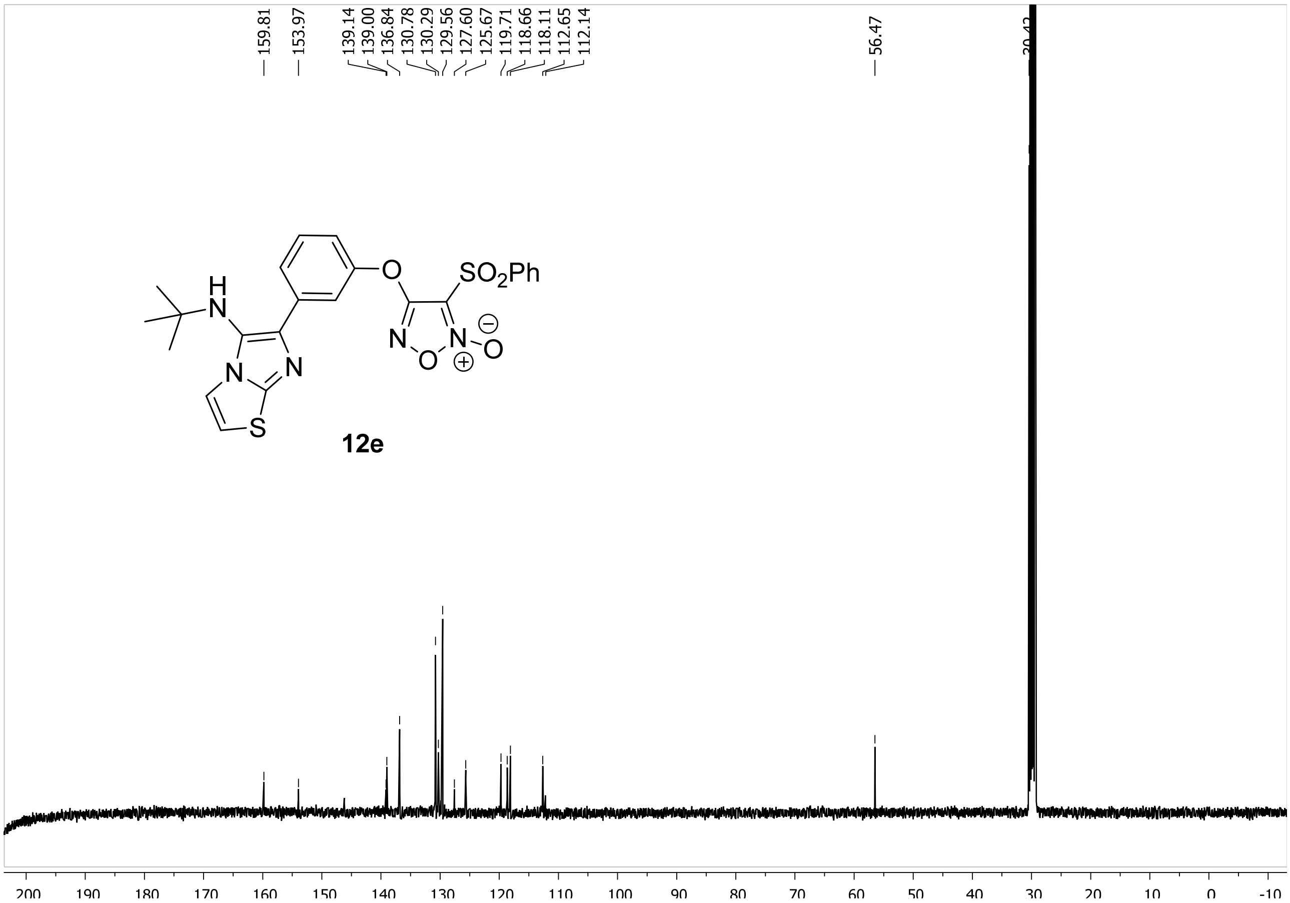
159.81
153.97
139.14
139.00
136.84
130.78
130.29
129.56
127.60
125.67
119.71
118.66
118.11
112.65
112.14
56.47
H
N
O
SO2Ph
N
N
O
O
N
N
S
12e
200 190 180 170 160 150 140 130 120 110 100 90 80 70 60 50 40 30 20 10 0 -10
